# Type 2 Diabetes as a Determinant of Parkinson’s Disease Risk and Progression

**DOI:** 10.1002/mds.28551

**Published:** 2021-03-08

**Authors:** Harneek Chohan, Konstantin Senkevich, Radhika K. Patel, Jonathan P. Bestwick, Benjamin M. Jacobs, Sara Bandres Ciga, Ziv Gan-Or, Alastair J. Noyce

**Affiliations:** 1 Preventive Neurology Unit, Wolfson Institute of Preventive Medicine, Barts and the London School of Medicine and Dentistry, Queen Mary University of London, London, UK; 2 Department of Neurology and Neurosurgery, McGill University, Montréal, Quebec, Canada; 3 Montreal Neurological Institute, McGill University, Montréal, Quebec, Canada; 4 Molecular Genetics Section, Laboratory of Neurogenetics, National Institute on Aging, National Institutes of Health, Bethesda, Maryland, USA; 5 Department of Human Genetics, McGill University, Montréal, Quebec, Canada; 6 Department of Clinical and Movement Neurosciences, UCL Institute of Neurology, London, UK

**Keywords:** Parkinson’s disease, type 2 diabetes

## Abstract

**Background:**

Type 2 diabetes (T2DM) and Parkinson’s disease (PD) are prevalent diseases that affect an aging population. Previous systematic reviews and meta-analyses have explored the relationship between diabetes and the risk of PD, but the results have been conflicting.

**Objective:**

The objective was to investigate T2DM as a determinant of PD through a meta-analysis of observational and genetic summary data.

**Methods:**

A systematic review and meta-analysis of observational studies was undertaken by searching 6 databases. We selected the highest-quality studies investigating the association of T2DM with PD risk and progression. We then used Mendelian randomization (MR) to investigate the causal effects of genetic liability toward T2DM on PD risk and progression, using summary data derived from genome-wide association studies.

**Results:**

In the observational part of the study, pooled effect estimates showed that T2DM was associated with an increased risk of PD (odds ratio [OR] 1.21, 95% confidence interval [CI] 1.07–1.36), and there was some evidence that T2DM was associated with faster progression of motor symptoms (standardized mean difference [SMD] 0.55, 95% CI 0.39–0.72) and cognitive decline (SMD −0.92, 95% CI −1.50 to −0.34). Using MR, we found supportive evidence for a causal effect of diabetes on PD risk (inverse-variance weighted method [IVW] OR 1.08, 95% CI 1.02–1.14; *P* = 0.010) and some evidence of an effect on motor progression (IVW OR 1.10, 95% CI 1.01–1.20; *P* = 0.032) but not on cognitive progression.

**Conclusions:**

Using meta-analyses of traditional observational studies and genetic data, we observed convincing evidence for an effect of T2DM on PD risk and new evidence to support a role in PD progression.

Type 2 diabetes (T2DM) and Parkinson’s disease (PD) are prevalent diseases that affect an aging population. Emerging evidence suggests biological relationships between the two. Both are characterized by aberrant protein accumulation, lysosomal and mitochondrial dysfunction, and chronic systemic inflammation.^1,2^ Insulin resistance is a hallmark of T2DM and may be an important contributing factor to PD too.^3^

Previous systematic reviews and meta-analyses have explored the relationship between diabetes and the risk of PD, but the results are conflicting. For instance, pooled effect estimates from case–control studies suggest that diabetes has a negative association with PD risk,^4–6^ whereas meta-analyses that focus on prospective cohort studies suggest an increased risk of PD in patients with diabetes.^5,7^ Importantly, these analyses included patients with diabetes, in general, rather than T2DM, specifically, and have not considered the effect T2DM has on the progression of PD.

The association between T2DM and risk of PD has also not been explored thoroughly using modern causal methods. Mendelian randomization (MR) is a method in genetic epidemiology that can be used to follow up observational associations for evidence of true causal effects.^8^ Genetic variants are distributed randomly at birth; therefore, the genetic determinants of an exposure (here T2DM) are not affected by the presence of the outcome (here PD). The current study combines the meta-analysis of observational data with that of genetic data (MR) to evaluate the effect T2DM has on the risk of developing PD and on motor and cognitive progression in patients with PD.

## Patients and Methods

### Observational Study Data

#### Literature Searches

The Meta-Analyses of Observational Studies in Epidemiology guidelines were followed to conduct the literature search. Various electronic databases, including PubMed, Web of Science, Scopus, and Ovid, were used. In addition, the preprint electronic servers bioRxiv and medRxiv were used for a more comprehensive search of recent literature. The searches took place between June 2, 2020, and June 6, 2020.

The terms used for searches were “Type 2 Diabetes” AND “Parkinson’s disease” OR “Type 2 Diabetes” AND “progression of Parkinson’s disease” OR “Type 2 diabetes” AND “Parkinson’s disease risk.” The reference lists of included articles were hand-searched to ensure the inclusion of any missed articles. Independent literature searches were conducted by two authors (H.C. and R.K.P.).

#### Inclusion Criteria

The inclusion of studies for the main meta-analysis consisted of (1) observational studies that investigated preceding T2DM specifically and its effect on the risk of PD and (2) studies analyzing how diabetes was associated with the progression of PD. Observational studies of the association between diabetes (in general) and risk of PD were analyzed separately.

#### Exclusion Criteria

**FIG. 1.**
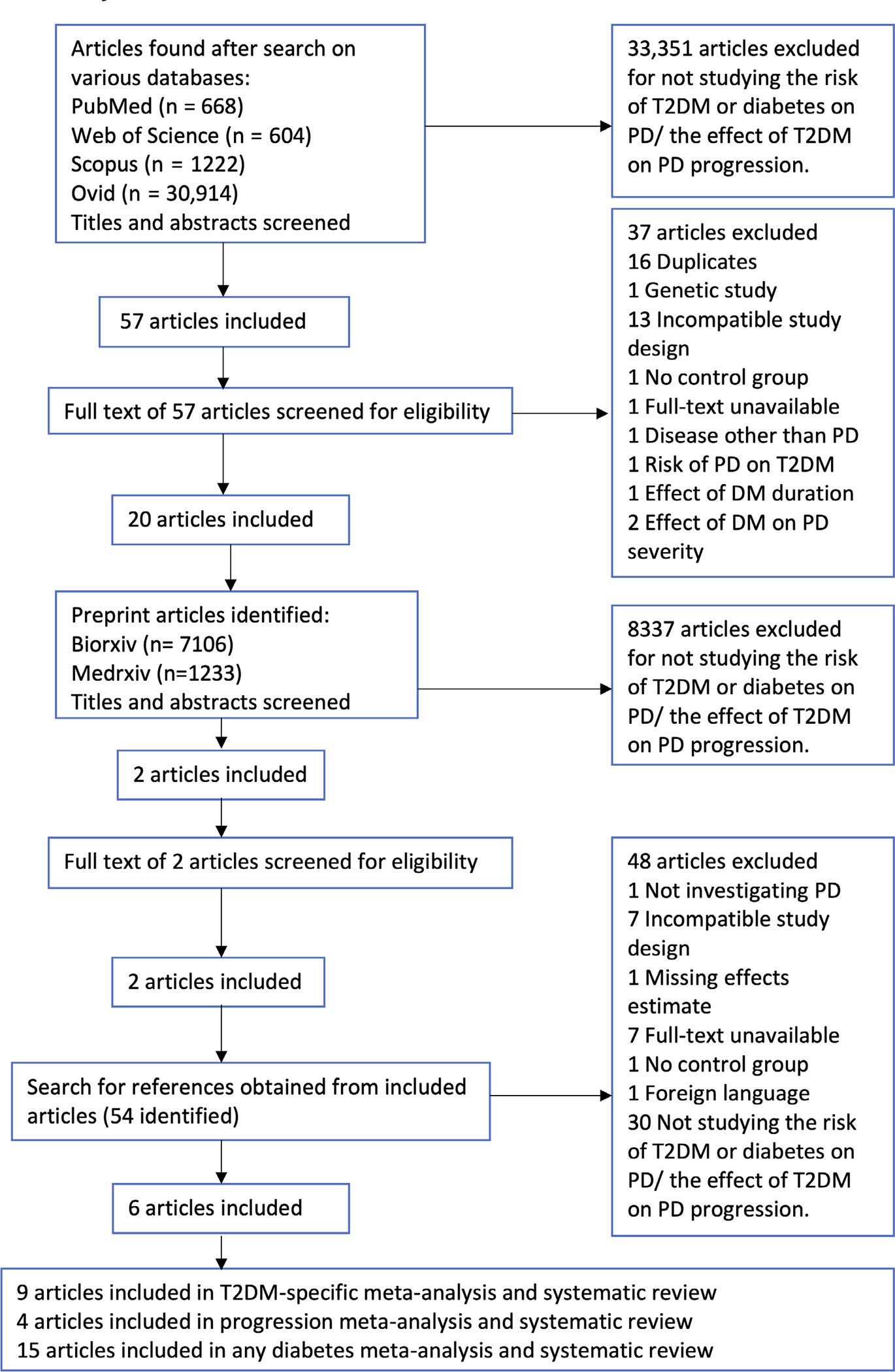
PRISMA flowchart used for the systematic review process. T2DM, type 2 diabetes mellitus; PD, Parkinson’s disease; DM, diabetes mellitus; PRISMA, Preferred Reporting Items for Systematic Reviews and Meta-Analyses. [Color figure can be viewed at wileyonlinelibrary.com]

Articles that were not observational studies, such as reviews, short surveys, books, and letters that reported no new data, were excluded (see Fig. 1). Any non-English articles were excluded.

#### Data Extraction

The Preferred Reporting Items for Systematic Reviews and Meta-Analyses guidelines were followed for data extraction.^9^ The data extracted from the articles included the study design, the effect estimates and the 95% confidence intervals (CI), and any adjustments made for confounding factors. For studies reporting data for men and women separately, the effect estimates for both sexes combined were used. The Newcastle Ottawa Scale (NOS) was used to assess the quality of the studies included.^10^

#### Statistical Analysis

Effect estimates from individual studies of PD risk were used to calculate a combined effect estimate for the association between T2DM and risk of PD using standard meta-analysis methods. Effect estimates from case–control and cohort studies were meta-analyzed separately and then together. For PD progression, we used standard motor scales (part III of the Unified Parkinson’s Disease Rating Scale [UPDRS] or Hoehn and Yahr stage) and cognitive scales (MoCA [Montreal Cognitive Assessment] or MMSE [Mini-Mental State Exam]). The mean progression per year was calculated to generate a standardized mean difference (SMD) for each study on a 1-year scale. For studies providing a ratio effect estimate, the ratio was also converted into a SMD.^11^ For all meta-analyses, the effect estimates were pooled using fixed effects and weighted by inverse variance before being assessed for heterogeneity using the I^2^ statistic. Where there was evidence of heterogeneity (I^2^ > 50% and *P* < 0.05), the meta-analysis was rerun using a random-effects model to combine effect estimates. As a result of these steps, a random-effects model was used to combine estimates for risk and a fixed-effects model was used for progression. To assess reasons for heterogeneity and to check for bias that might have explained the differences in estimates in the studies investigating PD risk, the studies were separated by study design and time of patient enrolment (patients enrolled prior to the exposure [T2DM] being observed, patients enrolled after the exposure had occurred, and studies enrolling patients before and after exposure occurred). Further sub-analyses investigated if the duration of T2DM was a determinant of PD risk, and we also considered studies that excluded participants with prior cardiovascular disease (CVD). Funnel plots were drawn to assess publication bias, and where evidence for this was available, a trim-and-fill analysis was performed. Meta-regression was performed to investigate the effect of gender and age on effect estimates. Stata version 16.0 was used to perform these analyses.

### Mendelian Randomization

MR utilizes single-nucleotide polymorphisms (SNPs) associated with an exposure of interest (here T2DM) to assess evidence of a causal effect between that exposure and a disease outcome (here PD). A valid MR analysis has three core assumptions: (1) the SNPs are robustly associated with the exposure; (2) they are not associated with confounding factors; and (3) there is no alternative (pleiotropic) pathway that the SNP influences the outcome, other than via the exposure.^12^

#### Exposure Instrument

A recent multiethnic meta-analysis of genome-wide association studies (GWAS) on T2DM was selected as exposure to construct a genetic instrument for liability toward T2DM.^13^ To avoid bias due to population differences, we selected only GWAS-significant SNPs (*P* < 5 × 10^−8^) from participants of European ancestry (total 425 SNPs; N patients = 148,726, N controls = 965,732). We performed clumping with standard parameters (clumping window of 10,000 kb, R^2^ cutoff 0.001) to exclude variants in linkage disequilibrium. The proportion of variability explained by genetic variants (R^2^) and the strength of association with the exposure (*F*-statistic) were calculated using standard methods.^14,15^

#### Outcome Data

For PD risk, we used the summary statistics from the latest PD GWAS meta-analysis.^16^ Because participants from UK Biobank (UKB) data were present in both the T2DM GWAS and PD GWAS, we used a subset of the PD GWAS summary statistics that excluded UKB and 23andMe participants (N patients = 15,056, N controls = 12,637).

For PD progression, we sought similar data to that from the observational study meta-analysis (ie, UPDRS Part III, MMSE, and MoCA scores) derived from GWASs of these PD progression traits as outcomes.^17^ The sample sizes for PD progression studies were calculated as means across all SNPs in GWAS summary statistics. Based on the following sample sizes (PD risk N = 27,693; UPDRS Part III N = 1398; MMSE N = 1329; MoCA N = 1000), we calculated power to detect an odds ratio (OR) of 1.2 for each outcome (https://sb452.shinyapps.io/power/).^18^

#### Statistical Analysis

MR analyses were performed using two-sample MR package in R.^19,20^ Steiger filtering was applied to exclude SNPs that explained more variance in outcome than in exposure.21 We then calculated pooled causal estimates using the inverse-variance weighted method (IVW), which is the MR method with the highest statistical power when core MR assumptions are upheld.^20^ A variety of sensitivity analyses were performed. MR-Egger is a low-power statistical test that can demonstrate how a net directional pleiotropic effect could bias the IVW through violation of the third MR assumption.^22^ The weighted median method assumes that at least 50% of the weight of the SNPs are valid and therefore allows up to 50% of the SNPs to be invalid. The Mendelian Randomization Pleiotropy RESidual Sum and Outlier (MRPRESSO) method is used to detect pleiotropic outlier SNPs and then adjust the pooled estimate if necessary.^23^ Cochran’s Q test for the IVW analysis and the intercept test from MR-Egger were performed to assess for evidence of heterogeneity, which may result from pleiotropy.^24^ Finally, we reran the IVW analysis for the effect of genetically estimated T2DM on PD risk, after excluding SNPs that were also associated with BMI,^25^ to reduce confounding from another known determinant of PD risk.^15^

## Results

### Observational Study Data

The literature search produced 33,408 articles; 28 were eligible for meta-analyses. Details regarding the exclusion of articles can be found in Figure 1. Details of the included studies can be found in Tables S1–S3. Quality assessment using the NOS (Tables S4–S6) showed that studies included in the risk and progression meta-analyses were generally of good quality.

#### T2DM and Risk of PD

**FIG. 2.**
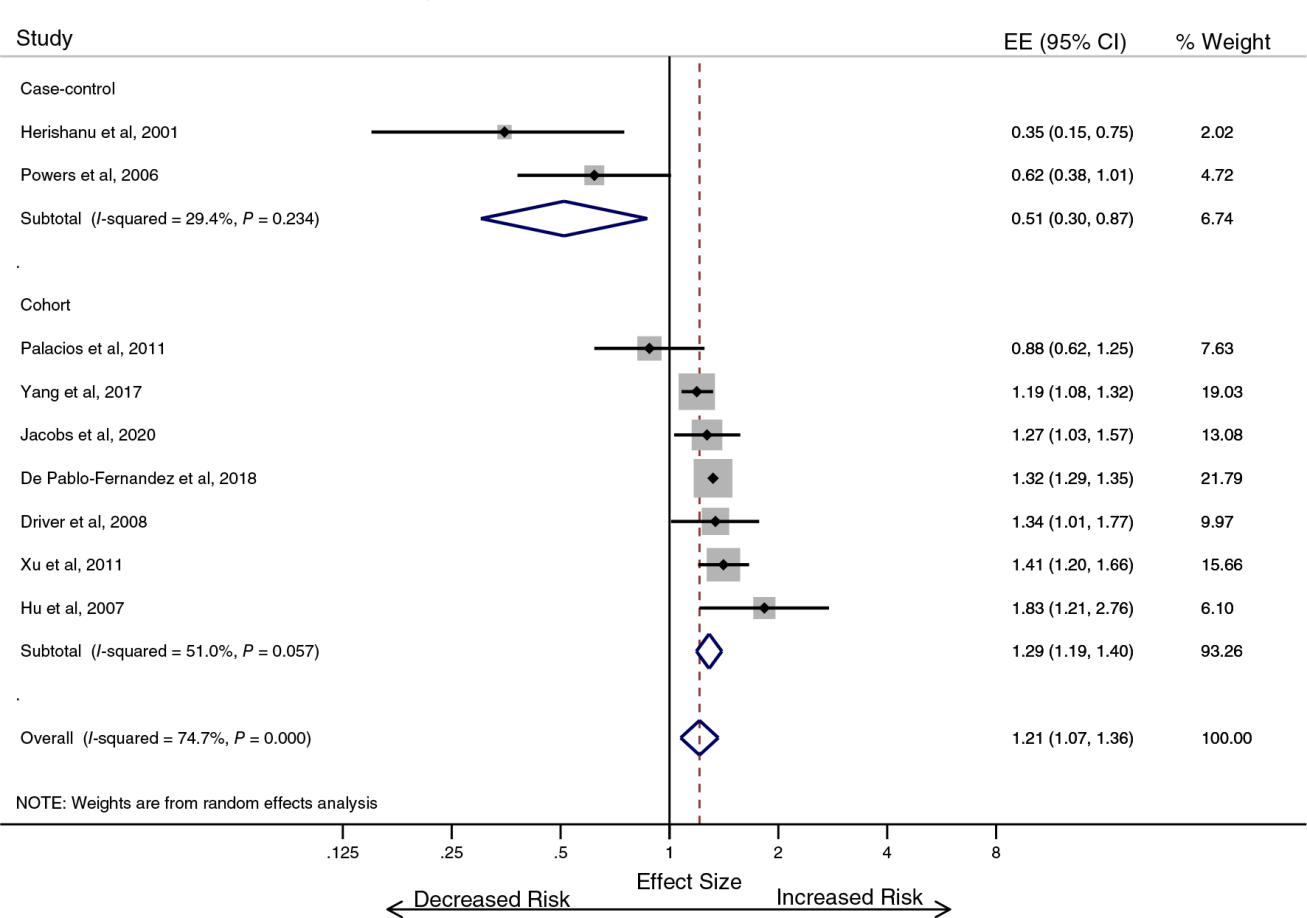
Type 2 diabetes is associated with an increase in the risk of developing Parkinson’s disease (T2DM-specific analysis). EE, effect estimate; CI, confidence interval. [Color figure can be viewed at wileyonlinelibrary.com]

From the nine studies that defined T2DM as the exposure,^26–34^ the pooled effect estimate was 1.21 (95% CI 1.07–1.36; see Fig. 2). The overall heterogeneity from the pooled analysis was high (I2 = 74.7%). Cohort studies (OR 1.29, 95% CI 1.19–1.40) provided strong evidence for T2DM being associated with higher PD risk, but there was an inverse association between T2DM and PD in case–control studies (OR 0.51, 95% CI 0.30–0.87). The funnel plot was visually asymmetric suggesting the possibility of publication bias (Fig. S1), but the accompanying *P*-value was 0.145.

The average age of participants in cohort studies was not a determinant of the association between T2DM and PD (*P* = 0.540; Fig. S2). Age was not investigated in the case–control studies due to there being only two. Similarly, the ratio of men and women did not account for differential study effects (*P* = 0.165). We then considered the cohort studies separately depending on when enrolment to the study occurred relative to the onset of the exposure (T2DM) under the assumption that the effect of survival bias in those with exposure before enrolment, unobserved) would be greatest.^35^ In one cohort study, the onset of T2DM was before the period of observation (OR 0.88, 95% CI 0.62–1.25), two of the cohort studies enrolled incident cases of T2DM (pooled OR 1.27, 95% CI 1.15–1.40), and the remaining four cohort studies used a combination of methods to determine the T2DM (pooled OR 1.38, 95% CI 1.24–1.55; Fig. S3).

Three studies from the main analysis provided information regarding the duration of T2DM.^31–33^ The pooled effect estimate from those three studies was 1.41 (95% CI 1.27–1.55), and heterogeneity was high (I^2^ = 89.7%). In patients with T2DM for less than 10 years, the effect was greater (OR 1.46, 95% CI 1.30–1.64) than it was for patients with T2DM for a duration of 10 years or more (OR 1.20, 95% CI 0.88–1.63). A separate analysis of the studies that specifically excluded participants with prior CVD also supported the notion that T2DM was associated with an increased risk of PD (OR 1.29, 95% CI 1.06–1.58; Fig. S4).

We repeated the risk meta-analysis using the studies that failed to specify T2DM as the exposure and included patients with *any* diabetes. These studies were generally lower in quality (Table S5),^36–49^ and heterogeneity was greater still (I^2^ = 88.4%). The combined effect estimate for all studies was 1.11 (95% CI 0.94–1.30), providing an overall null effect for the association between diabetes and risk of PD (Fig. S5). However, again there was a clear divergence in the pooled effect from case–control studies (OR 0.79, 95% CI 0.54–1.17) and cohort studies (OR 1.28, 95% CI 1.05–1.56). There was also evidence of publication bias in the combined analysis of case–control and cohort studies (*P* = 0.003; Fig. S5). The trim-and-fill sensitivity analysis (Fig. S7) led to an increase in the pooled effect estimate to 1.25 (95% CI 1.07–1.47), which was similar to the pooled effect in the main analysis. Meta-regression showed no evidence that the ratio of men to women was a determinant of the differential association between diabetes and PD risk across studies (*P* = 0.516). In case–control studies, as the average age of participants increased, the association with PD risk changed from negative to positive (*P* = 0.043; Fig. S8). There was no similar change in effect by participant age in the cohort studies (*P* = 0.268; Fig. S9). When case–control studies from the T2DM-specific and broader meta-analysis were pooled, there was no convincing effect of age (*P* = 0.053; Fig. S10).

#### Diabetes and Progression of PD

**FIG. 3.**
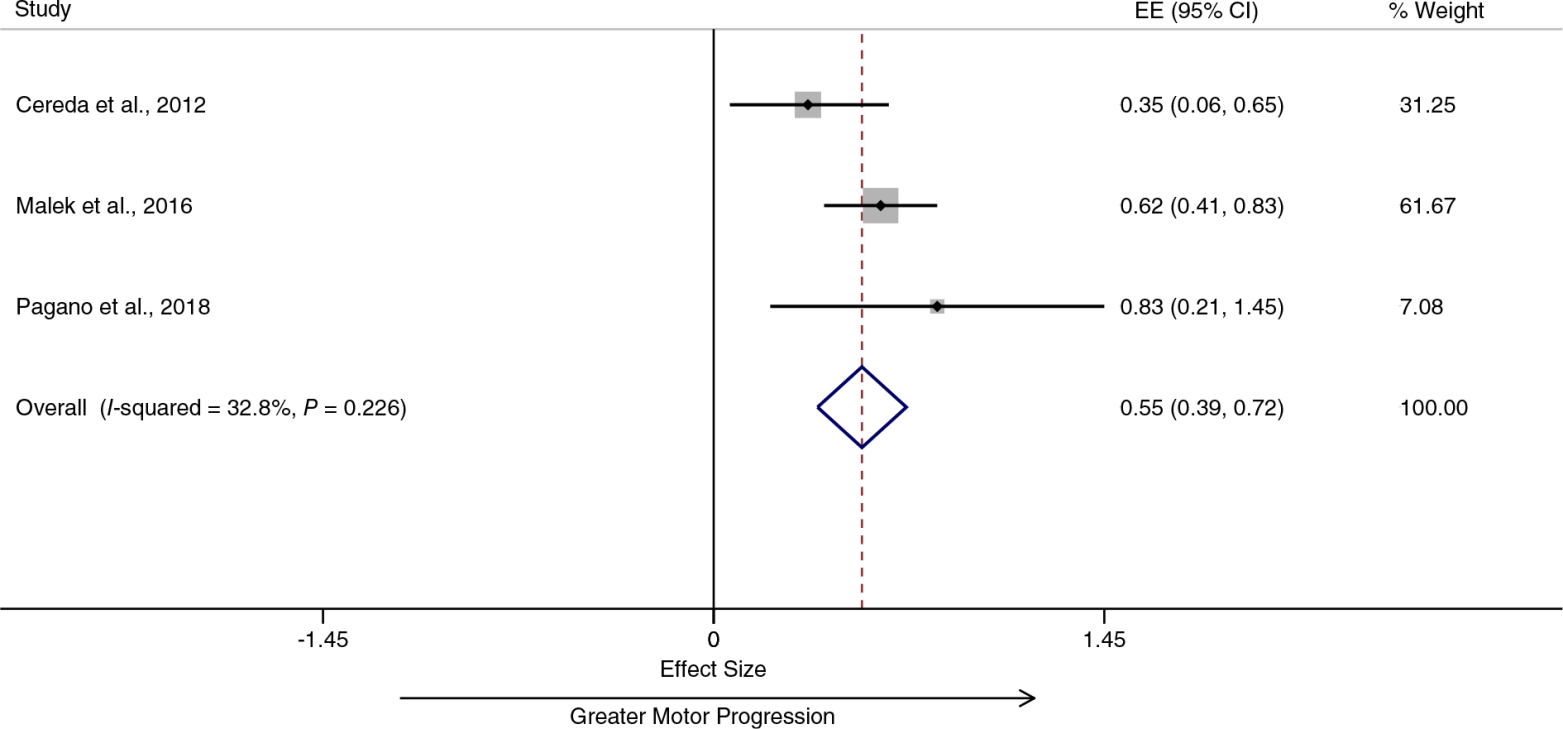
Diabetes is associated with greater motor progression in patients with Parkinson’s disease. EE, effect estimate; CI, confidence interval. [Color figure can be viewed at wileyonlinelibrary.com]

**FIG. 4.**
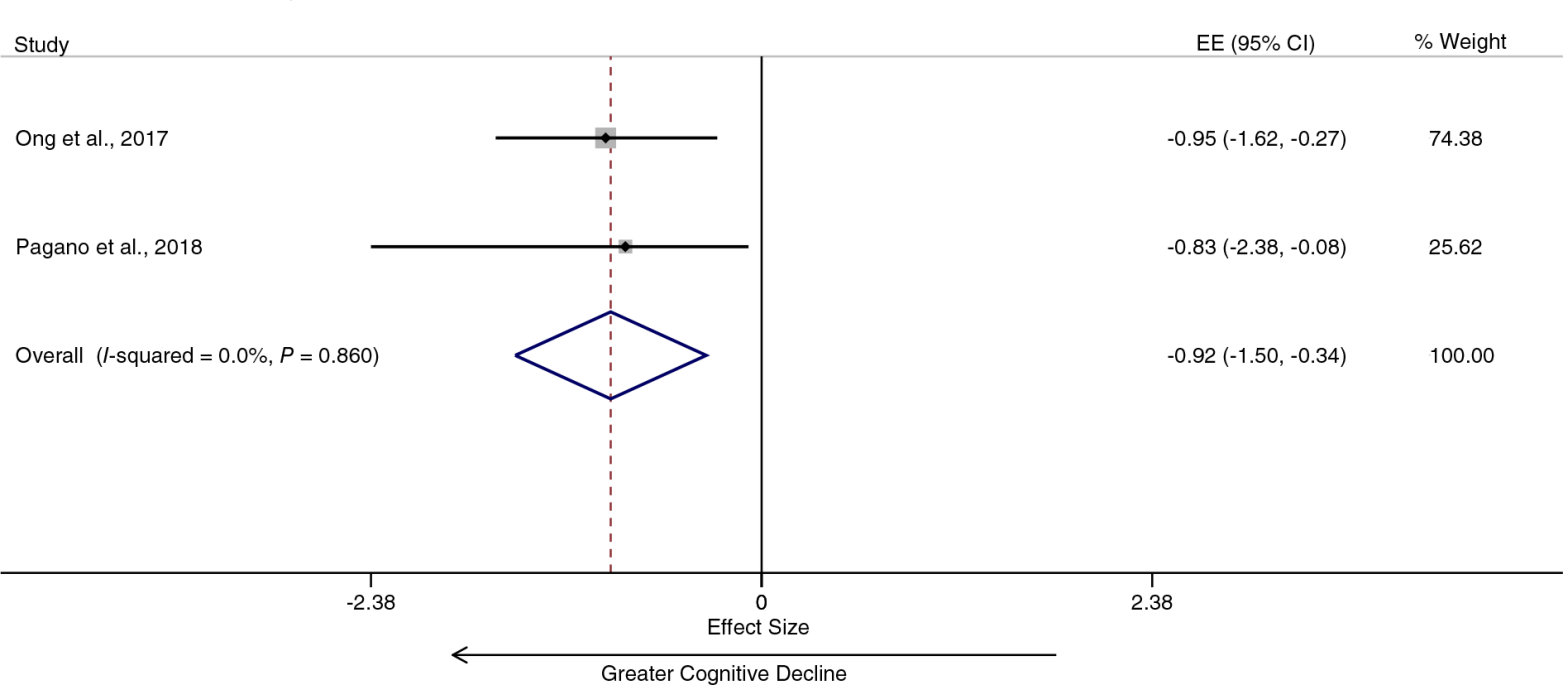
Diabetes is associated with greater cognitive decline in patients with Parkinson’s disease. EE, effect estimate; CI, confidence interval. [Color figure can be viewed at wileyonlinelibrary.com]

Pooling data from three studies provided evidence for diabetes being associated with faster progression in the severity of motor symptoms in PD patients^50–52^ (Fig. 3). The overall annual SMD was 0.55 (95% CI 0.39–0.72), and the heterogeneity was low (I^2^ = 32.8%). Pooling data from two studies provided evidence for diabetes also being associated with a faster cognitive decline in PD patients^52,53^ (Fig. 4). The overall annual SMD for cognitive change was −0.92 (95% CI −1.50 to −0.34), and there was no heterogeneity (I^2^ = 0.0%).

### Mendelian Randomization

Separately, we performed MR to assess evidence for a causal effect of T2DM on PD risk and progression. After clumping, the total number of independent SNPs that comprised the exposure instrument (liability toward T2DM) was 191. The instrument had a R^2^ = 1.5%, and the *F*-statistic was 51.2, with an *F*-statistic of 10 generally indicating an instrument of sufficient strength for causal analysis. We found evidence for a causal effect of liability toward T2DM on PD risk (IVW OR 1.08, 95% CI 1.02–1.14; *P* = 0.010; Table 1; Table S7; Figs. S11–S13). Sensitivity analyses, which explore different underlying assumptions, provided similar point estimates for the effect of genetically estimated T2DM on PD risk but with wider CIs, reflecting their lower statistical power (MR-Egger OR 1.05, 95% CI 0.93–1.17; *P* = 0.427; and weighted median OR 1.05, 95% CI 0.96–1.16; *P* = 0.299). The MR-PRESSO test to detect potential pleiotropic outlier SNP was nonsignificant (*P* = 0.990), and the pooled effect was therefore not adjusted further. We repeated the IVW analysis after excluding SNPs that were also associated with BMI and obtained very similar results to the IVW estimate, which included all T2DM SNPs (OR 1.09, 95% CI 1.01–1.17; *P* = 0.019).

There was some evidence for a causal effect of liability toward T2DM on PD progression measured using UPDRS, Part III (IVW OR 1.10, 95% CI 1.01–1.20; *P* = 0.032) but no convincing evidence for progression in the MoCA (IVW OR 0.81, 95% CI 0.49–1.33; *P* = 0.399) or MMSE scores (IVW OR = 0.99, 95% CI 0.85–1.14; *P* = 0.856). The directions of effect from all MR analyses were consistent with the effects from the observational study meta-analyses. The progression analyses, in particular, suffered from low statistical power.

## Discussion

In the present study, we used meta-analyses of observational and genetic data to investigate the role of T2DM as a determinant of both PD risk and progression. The results from the observational data meta-analyses and MR analyses were generally concordant; there was evidence from both methods that T2DM increases the risk of future PD. Our results also show that T2DM may increase the rate of motor progression of PD, with weaker support for an effect on cognitive progression.

The effect of T2DM on PD risk was clearest in the highest-quality prospective studies, which should be less affected by observer and selection bias. Unlike previous meta-analyses considering the association between diabetes and PD, we specifically considered T2DM for the main risk meta-analysis.^4–6^ When we relaxed the exposure definition to include all studies of diabetes, an overall null association between diabetes and risk of PD was found. We also observed in both risk analyses a now-recognized phenomenon of divergence in pooled effect estimates by study design, such that case–control studies tend to be associated with lower risk (even inverse risk) of PD and cohort studies with increased risk of PD.^2,4,5^

We extended observations about the effect of T2DM on PD risk to evaluate the limited available data on whether diabetes also affects PD progression. In data from observational studies, we observed evidence of an association between diabetes and PD progression. However, the evidence for this association being causal was observed only for an effect on PD motor scores in the MR part of the analysis, whereas there was an absence of evidence for an effect on PD cognitive progression. These progression MR analyses lacked statistical power relative to the risk MR analysis and all observational study analyses.

There is increasing evidence for shared biology between T2DM and PD. In T2DM, islet amyloid polypeptide (IAPP) or amylin aggregates to form amyloid plaques in pancreatic cells.^54^ Similarly, PD is pathologically defined by the accumulation of α-synuclein intraneuronally. Some evidence suggests that α-synuclein aggregation in PD occurs faster in the presence of IAPP.^55^ Circulating insulin may have a neuroprotective role, whereas systemic and local insulin resistance can influence pathways known to be important in PD pathogenesis, including those that relate to mitochondrial dysfunction, neuro-inflammation, and synaptic plasticity.^1,2^ Dopamine uptake is enhanced in the presence of insulin and dopamine release via enhanced cholinergic interneuron excitability, resulting in the activation of nicotinic acetylcholine receptors.^56^ The relationship between T2DM and neurodegeneration exists not only with PD. The AKT pathway is one of several insulin signaling pathways, and its overactivation has been related to aggravation of Alzheimer’s disease pathology.^57^ In PD, the AKT pathway is altered, causing an overexpression of GSK-3β that enhances the formation of neurofibrillary tangles, which may in turn contribute to PD dementia.^58^ As the global burden of T2DM increases sharply, it becomes increasingly important to understand its potential role in neurodegeneration.^59^

Associations between diabetes and PD tended to diverge by study design. Diabetes is associated with premature mortality, so an inverse association between diabetes and PD risk may occur as a result of greater midlife mortality in patients with diabetes.^6^ Survivor bias does not only affect case–control study designs, but also prospective cohorts in which the exposure occurs before the period of observation.^60^ We explored the potential role of survivor bias by examining case–control and cohort studies separately (discussed earlier), the effect of the average age of participants in studies, the gender ratio and by considering cohort studies separately depending on when the period of observation started relative to the onset of the exposure (T2DM). Average age and gender ratio had no major impact on observational study estimates. When cohort studies were separated according to the period of observation relative to the onset of the exposure, there were potentially revealing findings. One cohort study where the onset of T2DM was before the period of observation suggested a negative association with risk of PD but had wide CIs that crossed the null. Two of the cohort studies enrolled incident cases of T2DM, and the pooled effect was a precise estimate in favor of an increased risk of PD. The remaining cohort studies used mixed exposure ascertainment (self-reported history of diabetes at enrolment, ie, *unobserved;* and new cases, ie, *observed)*. The pooled point estimate from these studies was similar to the point estimate when the exposure was fully observed, but the CIs were wider. Overall, these sub-analyses indicated that only the highest-quality studies tend to support the association between T2DM and PD risk, whereas poorer-quality studies and those prone to the influence of survivor bias may underestimate it. Further support for an effect of survivor bias comes from the sub-analysis that considered the duration of exposure to T2DM; a weaker association with risk of PD was observed in patients with 10 or more years of T2DM relative to patients with T2DM for less than 10 years.

Strengths of this study are the specific focus on the role of T2DM for most of the analyses and the use of observational and genetic data to draw out causal inferences about the effect on PD risk and progression. The limitations include the possibility of bias, generalizability, limited statistical power for the progression MR analyses, and being unable to consider the effect of treatment of T2DM and severity.

An important consideration is that the majority of the observational data and all of the genetic data were derived from patients of European ancestry. Therefore, the results cannot be readily generalized to all populations. People of South-Asian and African-Caribbean descent are at greatest risk of T2DM.^61^ Furthermore, the effect of T2DM on PD progression may differ by ethnicity. A cohort study showed the prevalence of diabetes was highest in Hispanics (30%), and the risk of mild cognitive impairment due to T2DM was 11% in Hispanics compared to 4.6% in non-Hispanics.^62^ Therefore, the prevalence of diabetes in different ethnic groups may, in part, account for differences in PD prevalence and phenotype.^63^

The findings from this study do not consider the effect of anti-diabetic drugs on PD risk and progression. The repurposing of drugs used to treat T2DM for PD has been a major driver of the interest between the two conditions. A double-blind, placebo-controlled trial showed that the GLP-1 analogue, Exenatide, may have an effect on reducing PD severity after a prolonged washout period, increasing the possibility of a disease-modifying effect.^64^ A recent cohort study from the same group reported evidence that patients with T2DM taking certain classes of drugs (GLP-1 receptor agonists and DPP4 inhibitors) were at lower risk of PD than patients taking other oral antidiabetic drugs.^65^

Our analysis does not fully consider the likely important role of T2DM severity. T2DM represents a continuum ranging from individuals who are unaware of their disease state, to those who are aware but do not comply with management and are uncontrolled, and those who are diet controlled, tablet controlled, or insulin controlled, These factors were not considered in our analyses, and future studies may use biomarkers of glycemic control to further study the effects of T2DM severity on PD.^66^ For example, a recent study in nondiabetic patients reported that midlife variability in glycemic control was associated with future PD.^35^ Finally, our analysis does not consider that T2DM might influence markers of PD progression (motor and cognitive scores) independently of a direct effect on PD, and diabetic neuropathy and cognitive impairment due to small vessel disease are also commonly observed in older patients with diabetes.

In conclusion, we observe convincing evidence from the highest-quality observational studies that T2DM is associated with an increased PD risk and some evidence that it may contribute to faster PD motor and cognitive progression. More studies are needed to explore the role of T2DM as a determinant of PD progression and the strategies to modify this effect. Future observational and genetic studies should include greater representation of minority ethnic groups, many of whom experience a greater burden of T2DM. Treating T2DM may slow down the progression of PD. Thus, careful screening for T2DM and early treatment of T2DM in PD patients may be advisable. Finally, survival bias may be an important contributor to inverse associations between risk factors that cause premature mortality and age-related conditions, and this possibility warrants specific study.

## Supplementary Material

Supplementary Information

## Figures and Tables

**TABLE 1.** MR analysis between exposure (T2DM) and outcomes (PD risk and progression)

Outcome	N, SNPs included	Power	Inverse variance weighted		MR-Egger		Weighted median		MR-PRESSO global *P*
			OR (95% CI)	*P*	OR (95% CI)	*P*	OR (95% CI)	*P*	

PD risk	185	38.00%	1.08 (1.02–1.14)	0.010	1.05 (0.93–1.17)	0.427	1.05 (0.96–1.16)	0.299	0.990
Continuous PD progression traits									
UPDRS3	157	14.80%	1.10 (1.01–1.20)	0.032	1.09 (0.93–1.27)	0.301	1.14 (0.98–1.33)	0.101	0.841
MMSE	161	14.30%	0.99 (0.85–1.14)	0.856	1.21 (0.92–1.60)	0.177	1.02 (0.77–1.35)	0.897	0.848
MoCA	114	11.80%	0.81 (0.49–1.33)	0.399	0.41 (0.13–1.29)	0.129	0.71 (0.33–1.51)	0.371	0.233

Abbreviations: MR-PRESSO, Mendelian Randomization Pleiotropy RESidual Sum and Outlier; OR, odds ratio; CI, confidence interval; *P*, *P*-value; PD, Parkinson’s disease; UPDRS3, Unified Parkinson’s Disease Rating Scale, Part III; MMSE, Mini-Mental State Examination; MoCA, Montreal Cognitive Assessment.

## Data Availability

All data analyzed and generated in this study are included in this published article and Supplementary Information.
