# Supplementary material for: Type 2 Diabetes as a Determinant of Parkinson’s Disease Risk and Progression
Source: Mov Disord. Author manuscript; Available in PMC 2022 Apr 19. (PMC9017318; doi:10.1002/mds.28551)

Supplementary table 1: Studies included in the T2DM-specific meta-analysis from the literature review.

Study	Study Design	Ethnicity of participants	Follow-Up (years)	Average Age (years)	Sample Size	Effect Estimate (95% Confidence Interval)	Quality Score	Confounding Factors
Herishanu et al. 2001	Case-Control	Middle-Eastern	-	-	93 case and 93 controls	0.35 (0.15, 0.75)	Poor	-
Powers et al. 2006	Case-Control	Caucasian	-	70	352 cases and 484 controls	0.62 (0.38, 1.01)	Fair	Age, smoking, education and ethnicity.
Palacios et al. 2011	Cohort	Caucasian	13	71.6	656	0.88 (0.62, 1.25)	Good	Age, smoking, education, BMI, physical activity, caloric intake, caffeine intake, pesticide, exposure, alcohol intake and diary intake.
Yang et al. 2017	Cohort	East-Asian	7.3	56	36,294 (T2DM patients); 108,882 (non-T2DM patients)	1.19 (1.08, 1.32)	Good	Age, gender, insurance premium, residential area, type of occupation, CCI scores, comorbidity of schizophrenia and bipolar disorder, flunarizine use, metoclopramide use and zolpidem use.
Jacobs et al. 2020	Cohort	Caucasian	12	62.7	501,682	1.27 (1.03, 1.57)	Good	Age, sex, townsend deprivation index at recruitment and ethnicity.
De Pablo-Fernandez et al. 2018	Cohort	Caucasian	-	50	2,017,115 (T2DM cohort); 7,173,208 (reference cohort)	1.32 (1.29, 1.35)	Good	Sex, calendar year of cohort entry, age, region of residence and quintile Index of Multiple Deprivation score of patients.
Driver et al. 2008	Cohort	Caucasian	23.1	73.1	21,841	1.34 (1.01, 1.77)	Good	Age and smoking status.

Xu et al. 2011	Cohort	Caucasian	-	66.7	1,565	1.41 (1.2, 1.66)	Fair	Baseline age, race, sex, smoking status, education, physical activity and BMI.
Hu et al. 2007	Cohort	Caucasian	18	48.8	51,552	1.83 (1.21, 2.76)	Good	Age, sex, study year, BMI, systolic BP, cholesterol, education, alcohol consumption, tea consumption, coffee consumption, cigarette smoking, leisure-time physical activity and education.

T2DM- type 2 diabetes mellitus, BMI- body mass index, BP- blood pressure, CCI- Charlson comorbidity index.

Supplementary table 2: Studies included in the any diabetes meta-analysis from the literature review.

Study	Study Design	Ethnicity of participants	Follow-up (years)	Average Age	Sample Size	Effect Estimate (95% Confidence Interval)	Quality Score	Confounding Factors
Miyake et al. 2010	Case-Control	East Asian	-	67.7	249 cases and 368 controls	0.38 (0.17, 0.79)	Poor	Sex, age, region of residence, pack-years of smoking, years of education, leisure-time exercise, BMI, dietary intake of energy, cholesterol, vitamin E, alcohol, and coffee and the dietary glycaemic index.
D'Amelio et al. 2009	Case-Control	Caucasian	-	66.7	318 cases, 318 controls	0.4 (0.2, 0.8)	Good	BMI, smoking habit, education and occupational status.
Kessler 1972	Case-Control	Caucasian	-	67.8	228 cases, 228 controls	0.58 (0.3, 1.1)	Poor	Age
Savica et al. 2012	Case-Control	Caucasian	-	71	202 cases, 202 controls	0.67 (0.31, 1.48)	Fair	Age, sex, cigarette smoking and coffee consumption.
Rugbjerg et al. 2009	Case-Control	Caucasian	-	73	13,695 cases, 68445 controls	1.10 (0.8, 1.5)	Fair	Chronic obstructive pulmonary disease and sex.
Schernhammer et al. 2011	Case-Control	Caucasian	-	72.2	1,931 cases, 9,651 controls	1.35 (1.1, 1.65)	Fair	Age, sex and chronic obstructive pulmonary disease.
Morano et al. 1994	Case-Control	Caucasian	-	68.2	74 cases and 148 controls	1.39 (0.63, 3.05)	Poor	-

Leibson et al. 2006	Cohort	Caucasian	-	70	202 (PD patients), 202 (reference cohort)	0.70 (0.4, 1.4)	Poor	-
Simon et al. 2007	Cohort	Caucasian	22.9	66.6	171,879	1.04 (0.74, 1.46)	Fair	Age and smoking status.
Grandinettei et al. 1994	Cohort	Caucasian	26	69.7	8,006	1.20 (0.67, 2.12)	Poor	Age
Kim et al. 2018	Cohort	East Asian	10	64.5	7,746	1.26 (1.19, 1.33)	Poor	-
Sun et al. 2012	Cohort	East Asian	-	-	603,416 (diabetic patients); 472,188 (non-diabetic cohort)	1.61 (1.56, 1.66)	Good	Age, sex, geographic area, urbanisation status, hypertension, hyperlipidaemia and cardiovascular disease.
Skeie et al. 2013	Cohort	Caucasian	-	67.3	212 (PD cohort), 175 (control cohort)	1.94 (0.82, 4.57)	Fair	Age
Becker et al. 2008	Cross-Sectional	Caucasian	-	-	3,637 cases, 3,637 controls	0.95 (0.8, 1.14)	Good	BMI, smoking, asthma, dementia, hypertension, ischemic heart disease, congestive heart failure, stroke/transient ischemic attack, arrhythmia, hyperlipidaemia, epilepsy, affective disorders, schizophrenia, and neurotic and somatoform disorders.

De Pablo-Fernandez et al. 2017	Cross-Sectional	Caucasian	-	73	79 cases, 4919 controls	0.19 (0.9, 3.98)	Poor	Age, sex, hypertension, dyslipidaemia, antidiabetic treatment, alcohol consumption, smoking status, BMI, presence of cerebrovascular disease and treatment with potential parkinsonism-inducing drugs.

PD- Parkinson's disease, BMI- body mass index.

Supplementary table 3: Studies included in the progression meta-analysis from the literature review.

Study	Study Design	Motor or Cognitive Progression	T2DM	Ethnicity of participants	Time Period (years)	Sample Size	SMD (95% Confidence Interval)	Quality Score	Confounding Factors
Cereda et al. 2012	Case-Control	Motor	Yes	Caucasian	3	89 cases, 89 controls	0.35 (0.06, 0.65)	Fair	-
Malek et al. 2016	Cohort	Motor	No	Caucasian	3.5	1,759	0.62 (0.41, 0.83)	Good	Age, gender, disease duration and all vascular risk factors.
Pagano et al. 2018	Case-Control	Motor<br><br>Cognitive	Yes	Caucasian	3	25 cases, 14 controls	0.83 (0.21, 1.45)<br><br>-0.83 (-2.38, -0.08)	Good	Sex, age, H&Y stage and MDS-UPDRS Part III score.
Ong et al. 2017	Cross-Sectional	Cognitive	No	Caucasian	3	12 cases, 65 controls	-0.95 (-1.62, -0.27)	Fair	-

T2DM- type 2 diabetes mellitus; SMD- standardised mean difference, H&Y- Hoehn and Yahr, MDS-UPDRS- Movement Disorder Society-Unified Parkinson's Disease Rating Scale.

Supplementary table 4: Newcastle Ottawa Scale quality assessment of studies investigating the effect of T2DM on PD risk.

	Selection				Comparability	Outcome			Quality Score
Study	Representativeness of exposed cohort	Selection of the non-exposed cohort from same source as exposed cohort	Ascertainment of exposure	Demonstration that outcome of interest was not present at start of study	Comparability of cohorts on the basis of the design or analysis controlled for confounders	Assessment of outcome	Sufficient follow-up	Adequacy of follow up cohorts	
De Pablo-Fernandez et al. 2018	Participants were truly representative of patients with T2DM and were excluded if they had PD.	Yes	Secure record- ICD-10 code E11 (diagnosed with T2DM) from the English National Hospital Episode Statistics	Yes	Sex, calendar year of cohort entry, age, region of residence, and quintile Index of Multiple Deprivation score of patients.	Record-linkage	Follow up not specified	No statement	Good
Hu et al. 2007	Participants were truly representative of patients with T2DM in Finland. 5 geographic areas of	Yes	Self-report questionnaire	Yes	Age, sex, study year, BMI, systolic BP, cholesterol, education, alcohol consumption, tea consumption, coffee consumption, cigarette smoking,	All patients diagnosed with PD according to the criteria set by the Institution, the diagnosis is	Yes- mean follow up of 18 years.	Complete follow-up of all the patients	Good

	Finland were covered.				leisure-time physical activity and education.	based on medical history, clinical examination. The diagnosis needs to be done by a consultant.			
Xu et al. 2011	Participants were truly representative of patients with T2DM via the National Institutes of Health-AARP Diet and Healthy Study	Yes	Self-report	Yes	Baseline age, race, sec, smoking status, education, physical activity and BMI.	Diagnosed by Doctor	Not specified	No statement	Fair
Driver et al. 2008	Somewhat representative because females with PD were not included.	Yes	Self-report questionnaire	Yes	Age and smoking status	Self-report questionnaire	Yes Mean-23.1 years	Complete follow-up of all the patients	Good

Yang et al. 2017	Participants were truly representative of patients with T2DM in Taiwan. Patients were found.	Yes	Taiwan's National Health Research Institutes Dataset	Yes	Age and comorbidities	Record-linkage	Yes Mean-7.3 years	Complete follow-up of all the patients	Good
Palacios et al. 2011	Participants were somewhat representative of patients with T2DM.	Yes	Self-report	Yes	Age smoking, education, BMI, physical activity, caloric intake, caffeine intake, pesticide exposure, alcohol intake and diary intake.	Neurologists contacted and medical records checked.	Yes Mean- 13 years	No statement	Good
Powers et al. 2006	Participants were truly representative of patients with PD.	Yes	The Group Health Cooperative Health Maintenance Organisation.	Yes	Age, smoking, education and ethnicity	Diagnosed by neurologists	Follow up not specified	No statement	Fair
Herishanu et al. 2001	Participants were truly representative of patients with PD.	Yes	Outpatient PD clinical of Soroka University Medical Centre.	Yes	No description	No description	Follow up not specified	No statement	Poor

Jacobs et al. 2020	Participants were truly representative of patients with PD.	Yes	Linked Hospital Episode Statistics ICD codes or self-report	No	Age, sex, Townsend deprivation index at recruitment and ethnicity.	No description	Yes Mean- 12 years	Complete follow-up of all the patients	Good

T2DM- type 2 diabetes mellitus, PD- Parkinson's disease; ICD-10- International Classification of Disease, Tenth Revision; BMI- body mass index; BP- blood pressure;

Supplementary table 5: Newcastle Ottawa Scale quality assessment of studies investigating the effect of any diabetes on PD risk.

	Selection				Comparability	Outcome			Quality Score
Study	Representative-ness of exposed cohort	Selection of the non-exposed cohort from same source as exposed cohort	Ascertainment of exposure	Demonstration that outcome of interest was not present at start of study	Comparability of cohorts on the basis of the design or analysis controlled for confounders	Assessment of outcome	Was follow-up long enough for outcomes to occur	Adequacy of follow up cohorts	
Miyake et al. 2010	Participants were truly representative of patients with DM in Japan.	Yes	Hospital	Yes	Sex, age, region of residence, pack-years of smoking, years of education, leisure-time exercise, BMI, dietary intake of energy, cholesterol, vitamin E, alcohol, coffee and the dietary glycaemic index.	Self-reporting Questionnaire	Follow up not specified	No statement	Poor
Leibson et al. 2006	Participants were truly representative of patients with PD.	Yes	Census	No	Not specified	No description	Not specified	Not specified	Poor

Skeie et al. 2013	Participants were truly representative of patients with PD.	Yes	Norwegian PakWest study	Yes	Age	Structured interview	Not specified	Not specified	Fair
Morano et al. 1994	Participants are somewhat of patients with PD.	Yes	Hospitals	No	Not specified	Not specified	Not specified	Not specified	Poor
Savica et al. 2012	Participants were truly representative of patients with PD	No	Rochester Epidemiology Project	Yes	Age, sex, cigarette smoking and coffee consumption	Record-linkage	Not specified	Not specified	Fair
Rugbjerg et al. 2009	Participants were truly representative of patients with PD in Denmark	No	Danish National Hospital Register	Yes	Chronic obstructive pulmonary disease and sex	Hospital register	Not specified	Not specified	Fair
Kessler 1972	Participants were truly representative of patients with PD	Yes	Commercial sources	No	Age	Structured interview	Not specified	Note specified	Poor
Grandinettei et al. 1994	Participants were somewhat of patients with PD	No	Medical records	No	Age	Not specified	Yes- 26 years	Not specified	Poor

Kim et al. 2018	Participants were truly representative of patients with PD in South Korea	Yes	National Health Insurance Database	No	Not specified	Health insurance claims	Yes- 10 years	Not specified	Poor
Schernhammer et al. 2011	Participants were truly representative of patients with PD	No	Danish Hospital Register	Yes	Age, sex and chronic obstructive pulmonary disease	Danish Hospital Register	Not specified	Not specified	Fair
De Pablo-Fernandez et al. 2017	Participants were truly representative of patients with PD	Yes	NEDICES study	Yes	Age, sex, hypertension, dyslipidaemia, antidiabetic treatment, alcohol consumption, smoking status, BMI, presence of cerebrovascular disease and treatment with potential parkinsonism-inducing drugs.	Self-report	Not specified	Not specified	Poor
Simon et al. 2007	Participants were somewhat representative of patients with PD	No	Nurses' Health Study	No	Age and smoking status	Self-reported history	Yes- 22.9 years	Not specified	Fair

Becker at al., 2008	Participants were truly representative of patients with PD	Yes	UK- based General Practice Research Database	Yes	BMI, smoking, asthma/COPD, dementia, hypertension, ischemic heart disease, congestive heart failure, stroke/transient ischemic attack, arrhythmia, hyperlipidaemia, epilepsy, affective disorders, schizophrenia, and neurotic and somatoform disorders.	Patient records	Not specified	Not specified	Good
D'Amelio et al. 2009	Participants were truly representative of patients with PD in Italy	Yes	Neurological Department of Palermo	No	BMI, smoking habit, education and occupational status	Semi-structured questionnaire	Not specified	Subjects lost to follow up unlikely to introduce bias- number lost less than 20%	Good

Sun et al. 2012	Participants were truly representative of patients with DM in Taiwan	Yes	NHI claim data of Taiwan	Yes	Age, sex, geographic area, urbanisation status, hypertension, hyperlipidaemia and cardiovascular disease.	Hospital records	Yes- 1 year	Not specified	Good

PD- Parkinson's disease; T2DM- type 2 diabetes mellitus; BMI- body mass index; COPD- chronic obstructive pulmonary disease; DM- disease mellitus.

Supplementary table 6: Newcastle Ottawa Scale quality assessment of studies investigating the effect of diabetes on PD progression.

	Selection				Comparability	Outcome			Quality Score
Study	Representativeness of exposed cohort	Selection of the non-exposed cohort from same source as exposed cohort	Ascertainment of exposure	Demonstration that outcome of interest was not present at start of study	Comparability of cohorts on the basis of the design or analysis controlled for confounders	Assessment of outcome	Sufficient follow-up	Adequacy of follow up cohorts	
Cereda et al. 2012	Participants were truly representative of patients with PD and T2DM.	Yes	The Parkinson Institute research database	Yes	No description	UPDRS scale	Yes- mean 3 years.	Subjects lost to follow up unlikely to introduce bias- number lost less than 20%	Fair
Malek et al. 2016	Participants were truly representative of patients with PD and T2DM.	Yes	Tracking Parkinson's study	Yes	All vascular risk factors	UPDRS scale	Yes- mean 2.6 years.	Subjects lost to follow up unlikely to introduce bias- number lost less than 20%	Good

Pagano et al. 2018	Participants were truly representative of patients with PD and T2DM.	Yes	Parkinson's Progression Markers Initiative database	Yes	Sex, age, H&Y stage and MDS-UPDRS Part III score	UPDRS scale and MoCA	Yes- 3 years.	No statement	Good
Ong et al. 2017	Participants were truly representative of patients with PD and T2DM.	Yes	No description	Yes	No description	MoCA	Yes- 36 months	No statement	Fair[1]

UPDRS- Unified Parkinson's Rating Scale; MoCA- Montreal Cognitive Assessment

Supplementary table 7: MR analysis between exposure (T2DM) and outcomes (PD-risk and progression).

Outcome	Heterogeneity tests						Test for directional horizontal pleiotropy		
	MR Egger			Inverse variance weighted			Egger_intercept	SE	p-value
	Q	Q_df	Q_pval	Q	Q_df	Q_pval			
PD risk	126.369	183.000	1.00	126.73	184	1.00	0.002	0.004	0.550
Continuous PD progression traits									
UPDRS3	140.270	156	0.81	140.29	157	0.83	0.001	0.005	0.885
MMSE	135.158	159	0.92	138.10	160	0.89	-0.015	0.009	0.088
MoCA	124.287	112	0.20	126.13	113	0.19	0.045	0.035	0.200

PD- Parkinson's disease; SE- standard error; UPDRS3- Unified Parkinson's Disease Rating Scale Part 3; MMSE- Mini Mental Stata Examination; MoCA- Montreal Cognitive Assessment.

Supplementary Figure 1: Funnel plot generated for T2DM-specific studies.

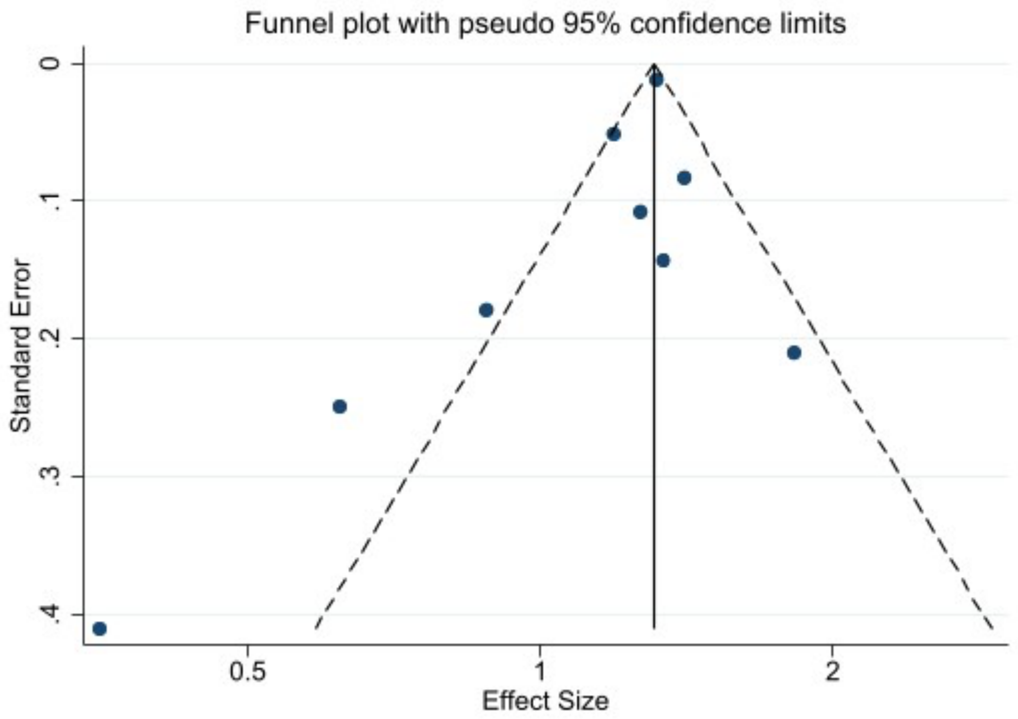

Supplementary Figure 2: PD risk decreases as average age of participants increases in T2DM-specific cohort studies.

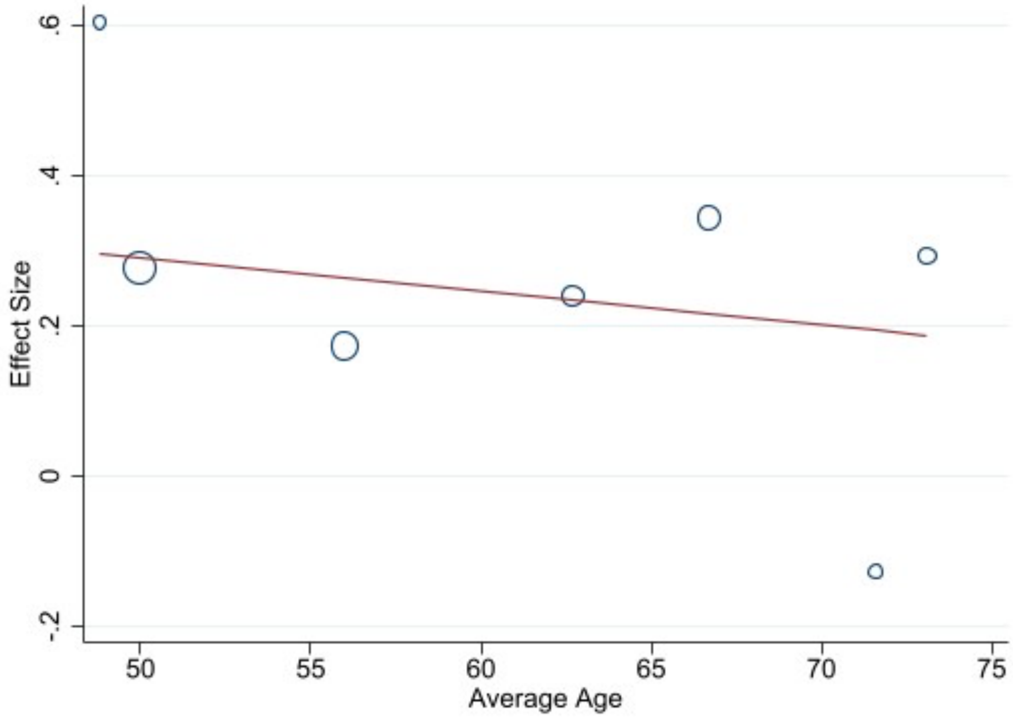

Supplementary figure 3: An observed exposure (T2DM) increases the risk of PD.

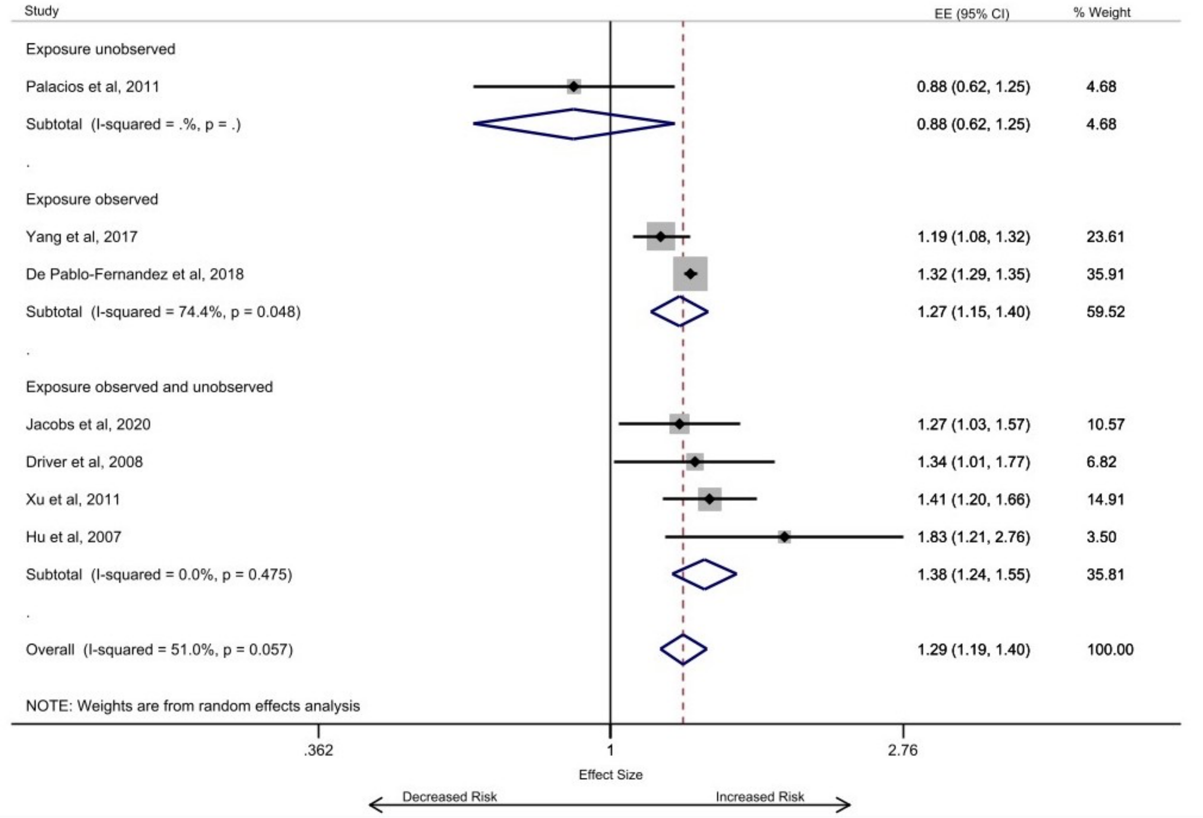

EE- effect estimate; CI- confidence interval.

Supplementary figure 4: Excluding patients with CVD further suggests T2DM is associated with an increased risk of PD.

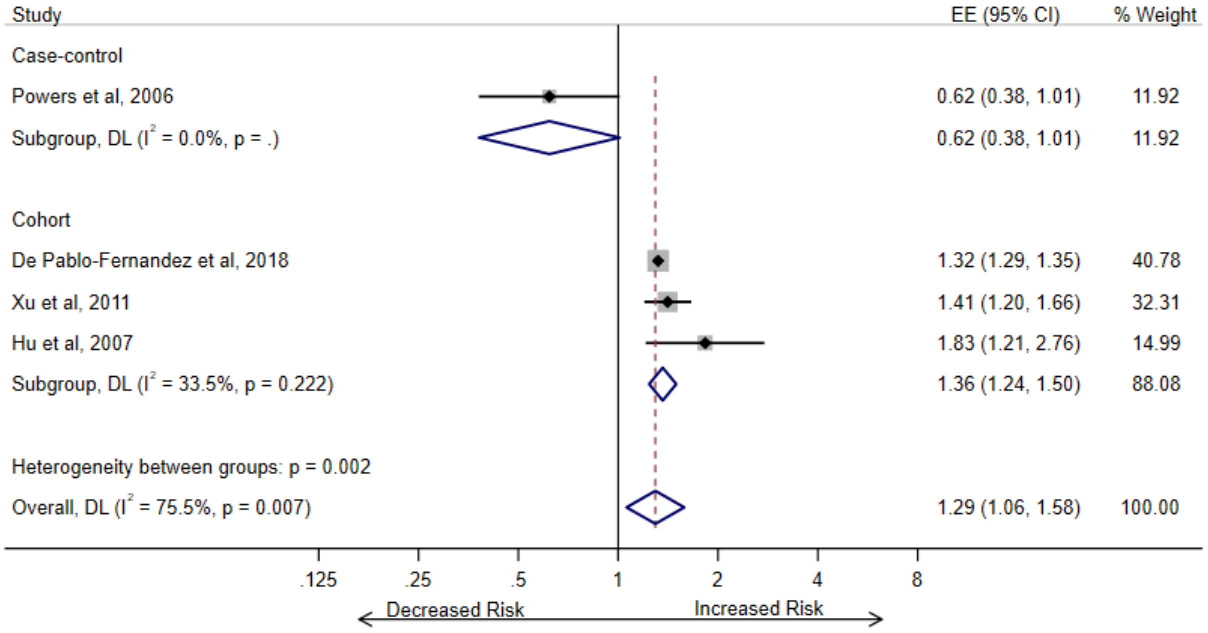

EE- effect estimate; CI- confidence interval.

Supplementary figure 5: Diabetes slightly increases the risk of Parkinson's disease (any diabetes).

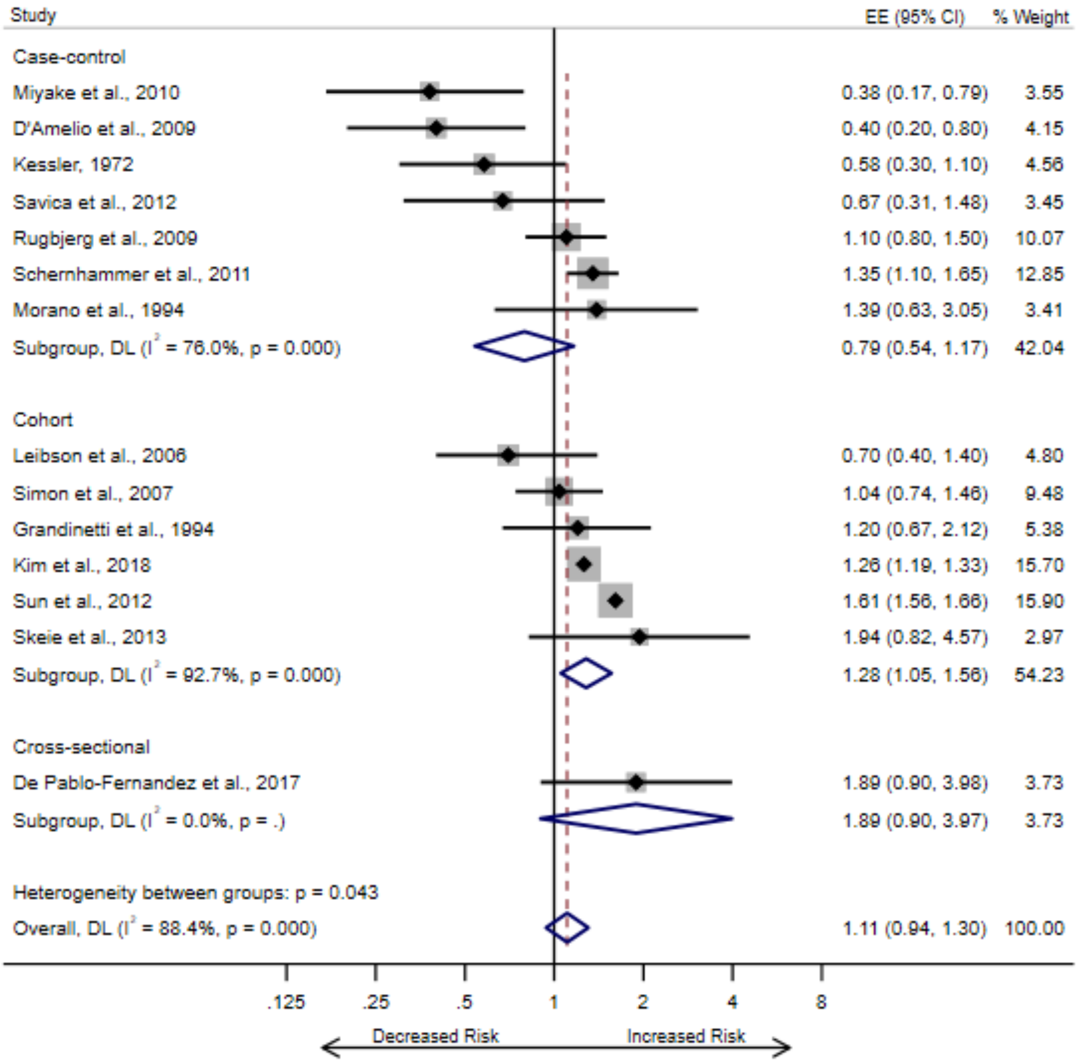

EE- effect estimate; CI- confidence interval.

Supplementary figure 6: Asymmetric funnel plot providing evidence for publication bias (any diabetes)

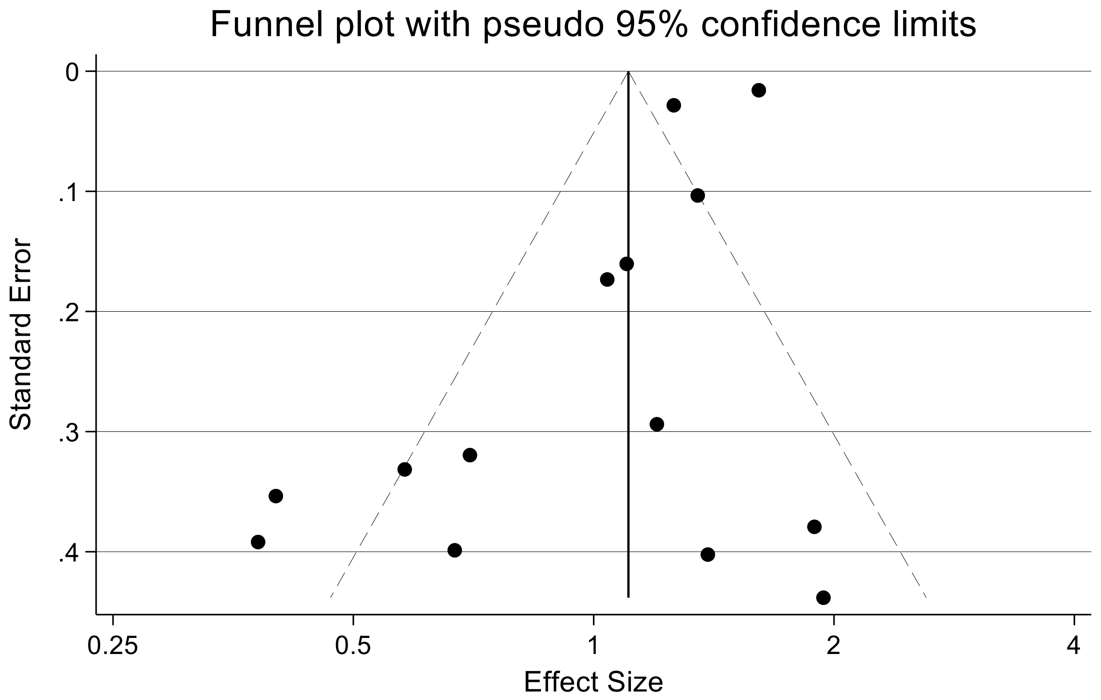

Supplementary figure 7: Funnel plot generated after trim and fill analysis to account for publication bias. Three studies were imputed.

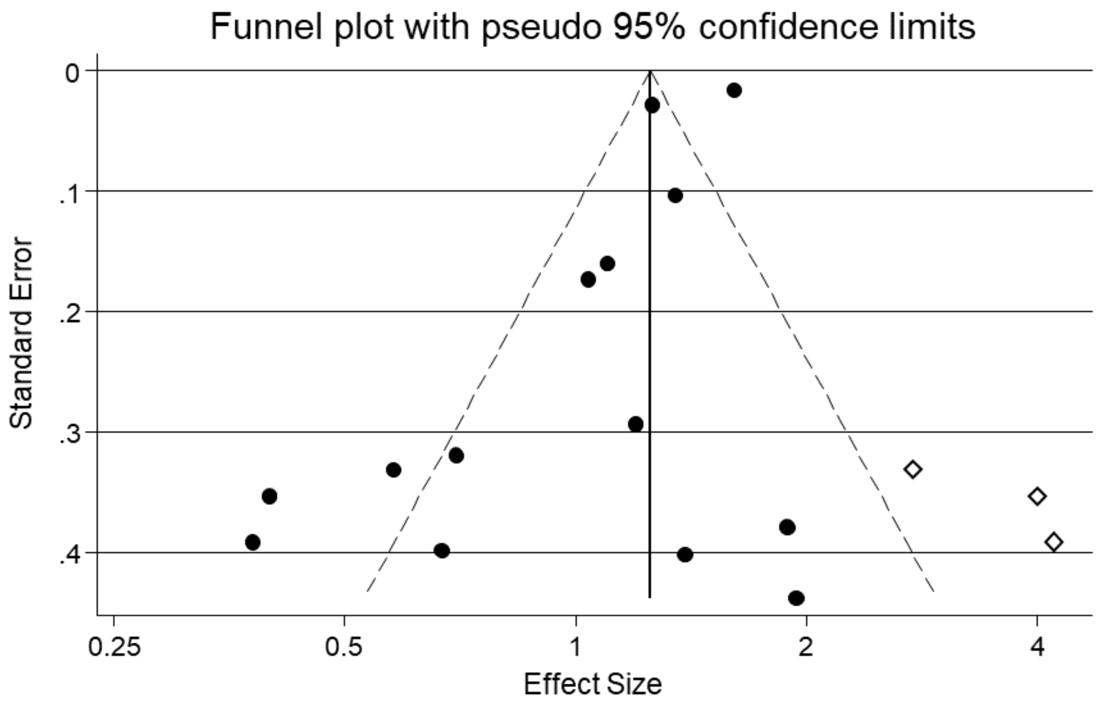

Supplementary figure 8: PD risk increases as average age of participants increases in case-control studies (any diabetes).

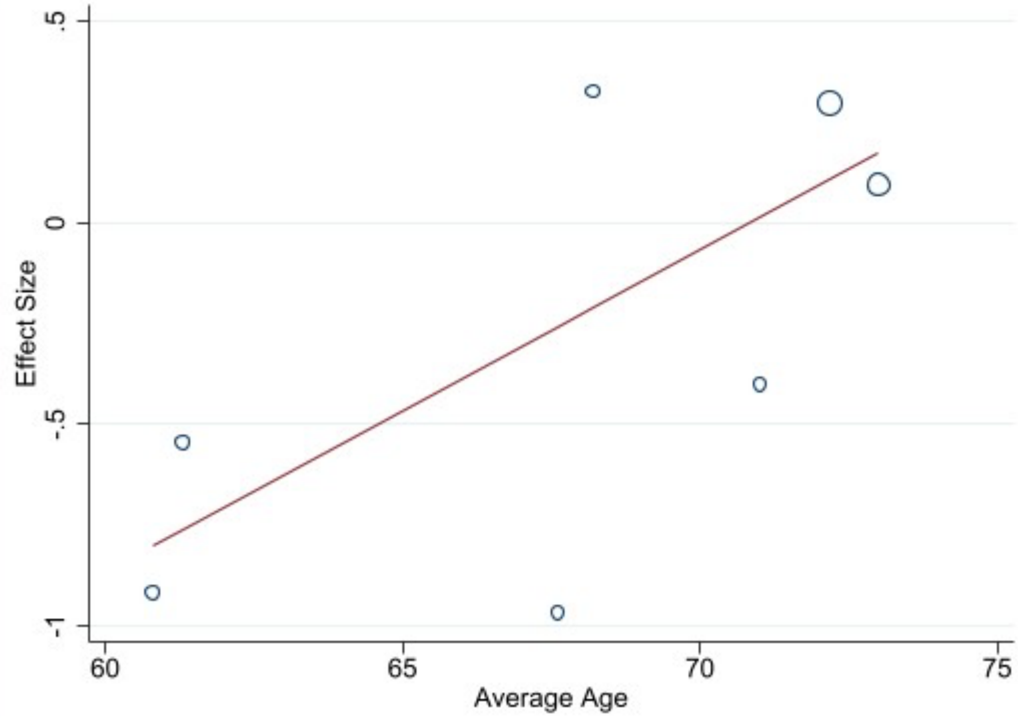

Supplementary figure 9: PD risk decreases as the average age of participants increases in cohort studies (any diabetes).

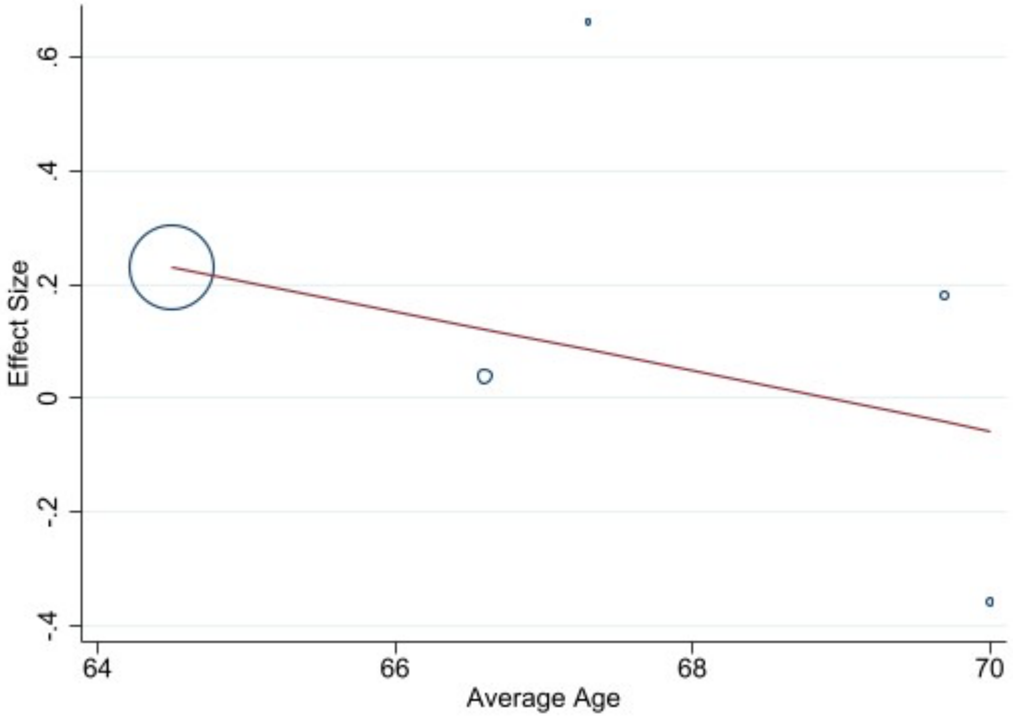

Supplementary figure 10: In the pooled case-control studies, as the average age of the participants increases the PD risk increases (T2DM-specific and any diabetes studies).

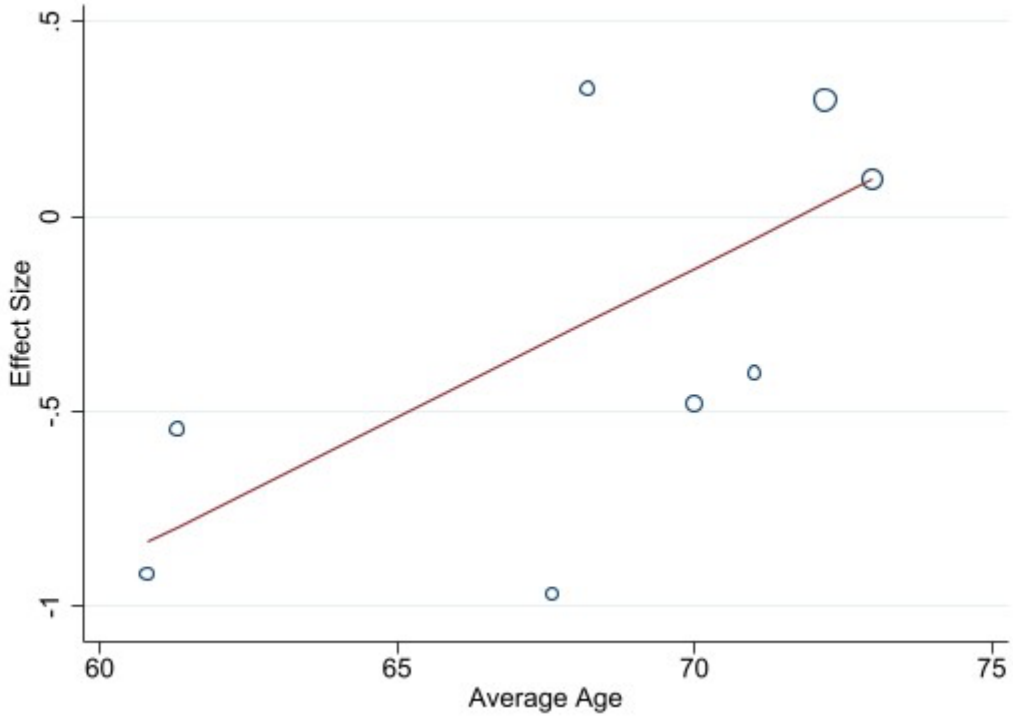

Supplementary Figure 11: Forest plots showing point estimates of the exposures of interest; Diabetes as exposure. PD risk and progression as outcomes.

PD risk as outcome

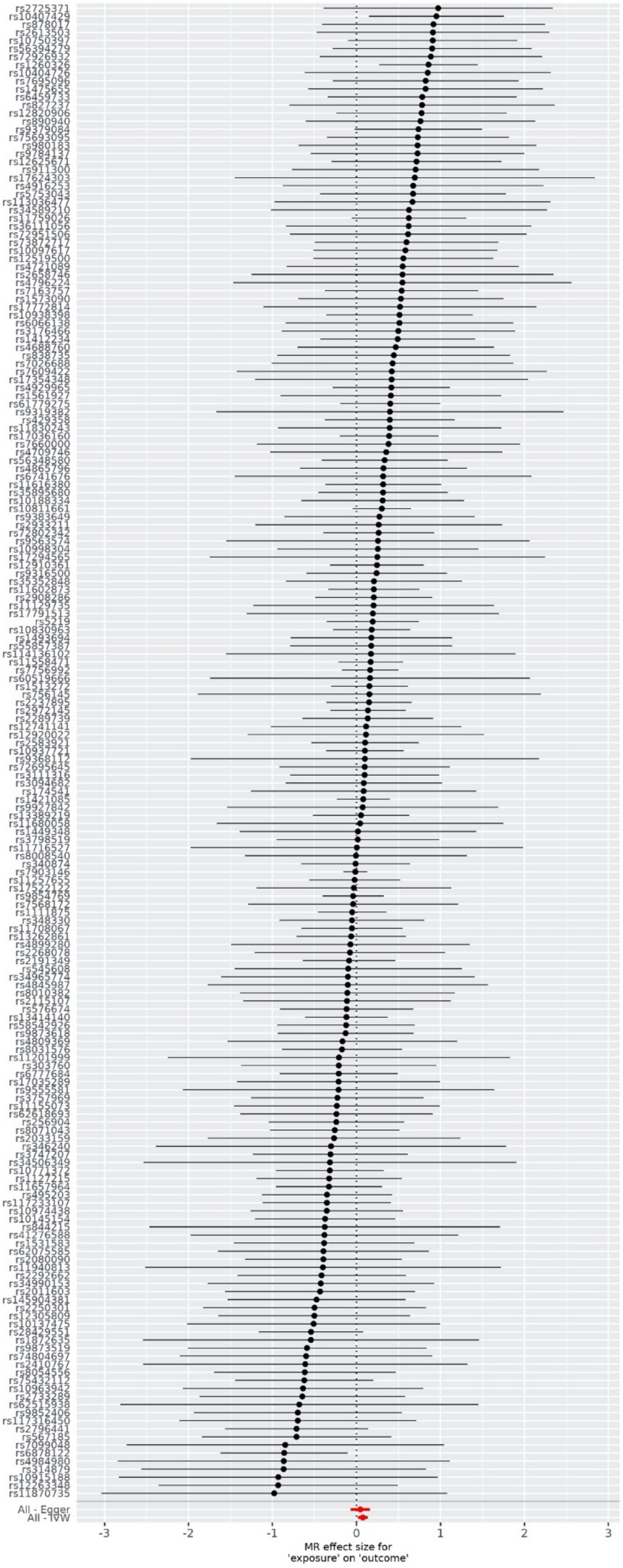

MMSE in PD as outcome

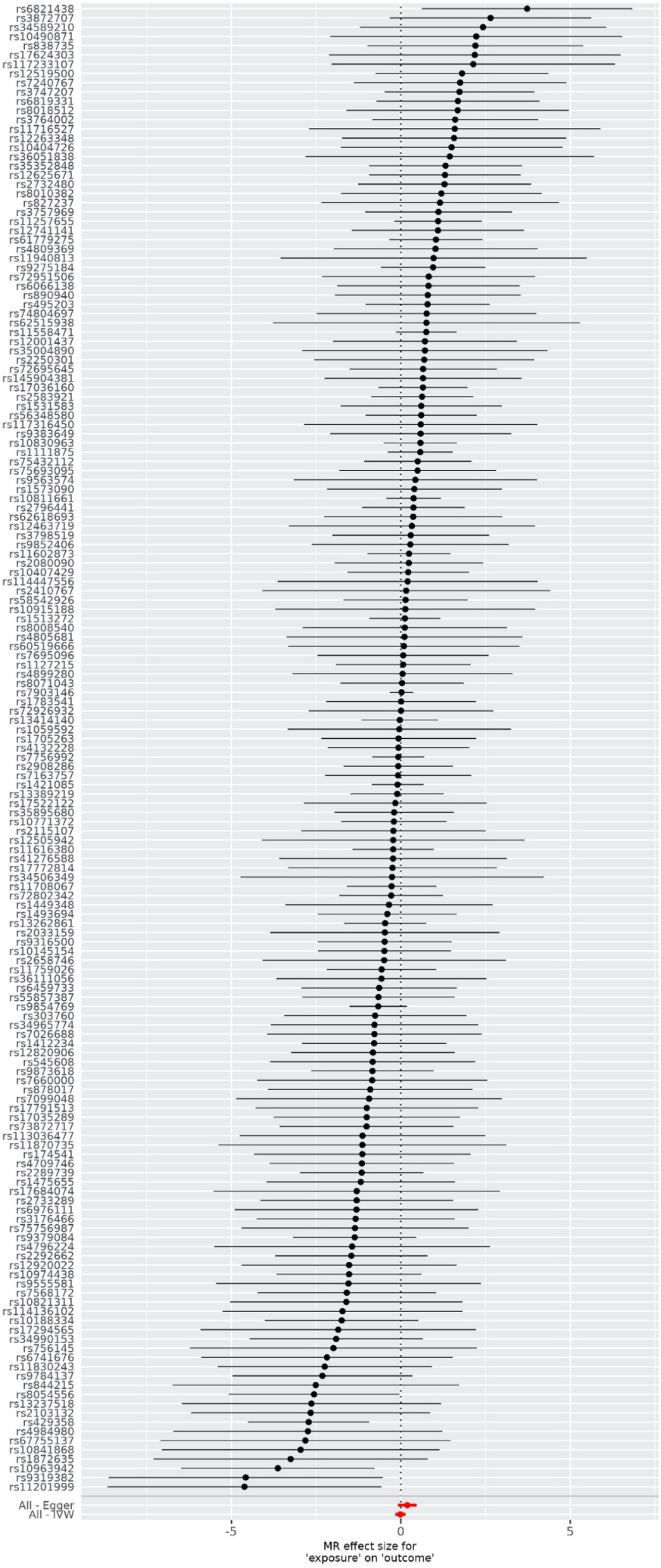

MoCA in PD as outcome

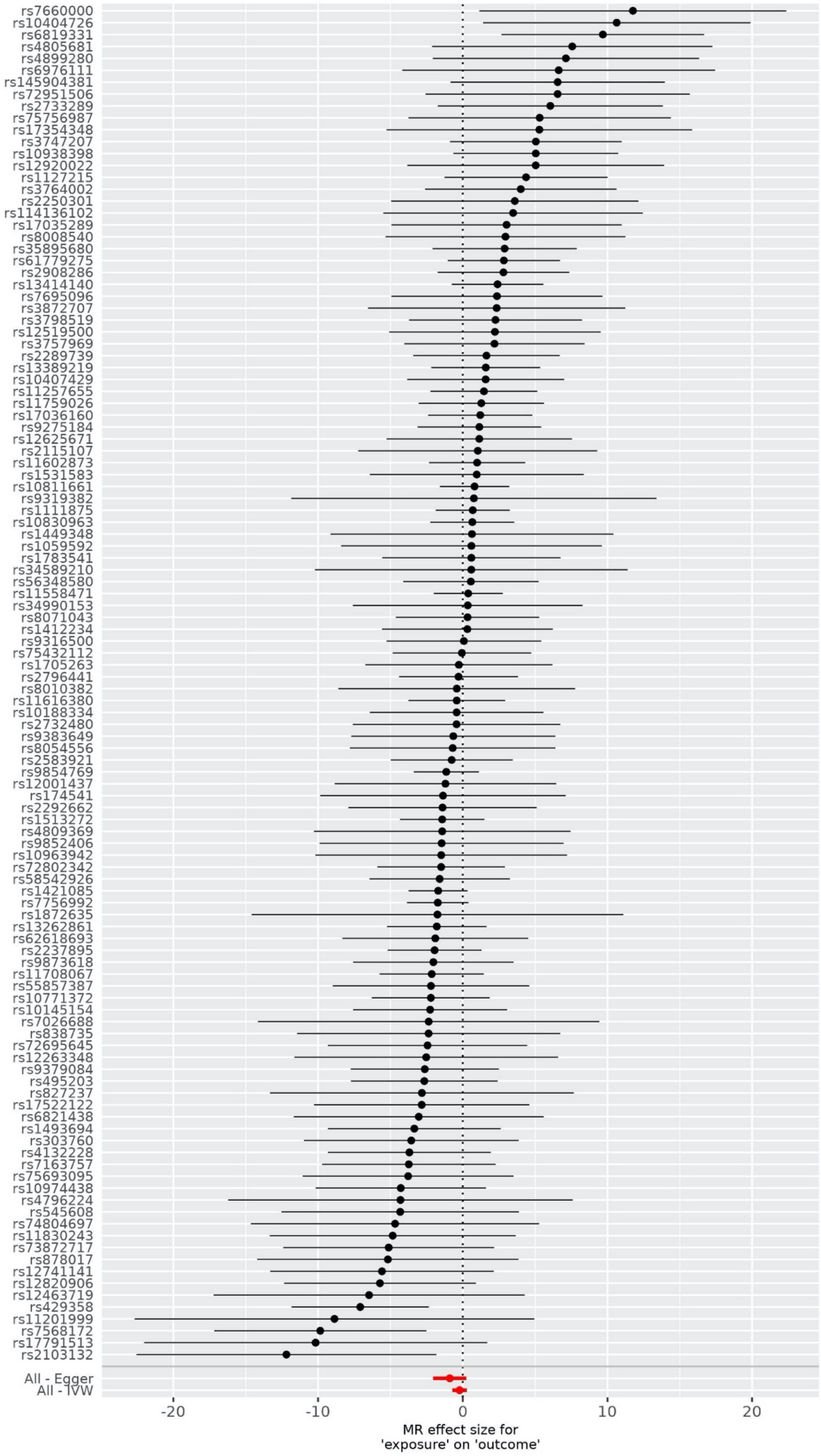

UPDRS3 in PD as outcome

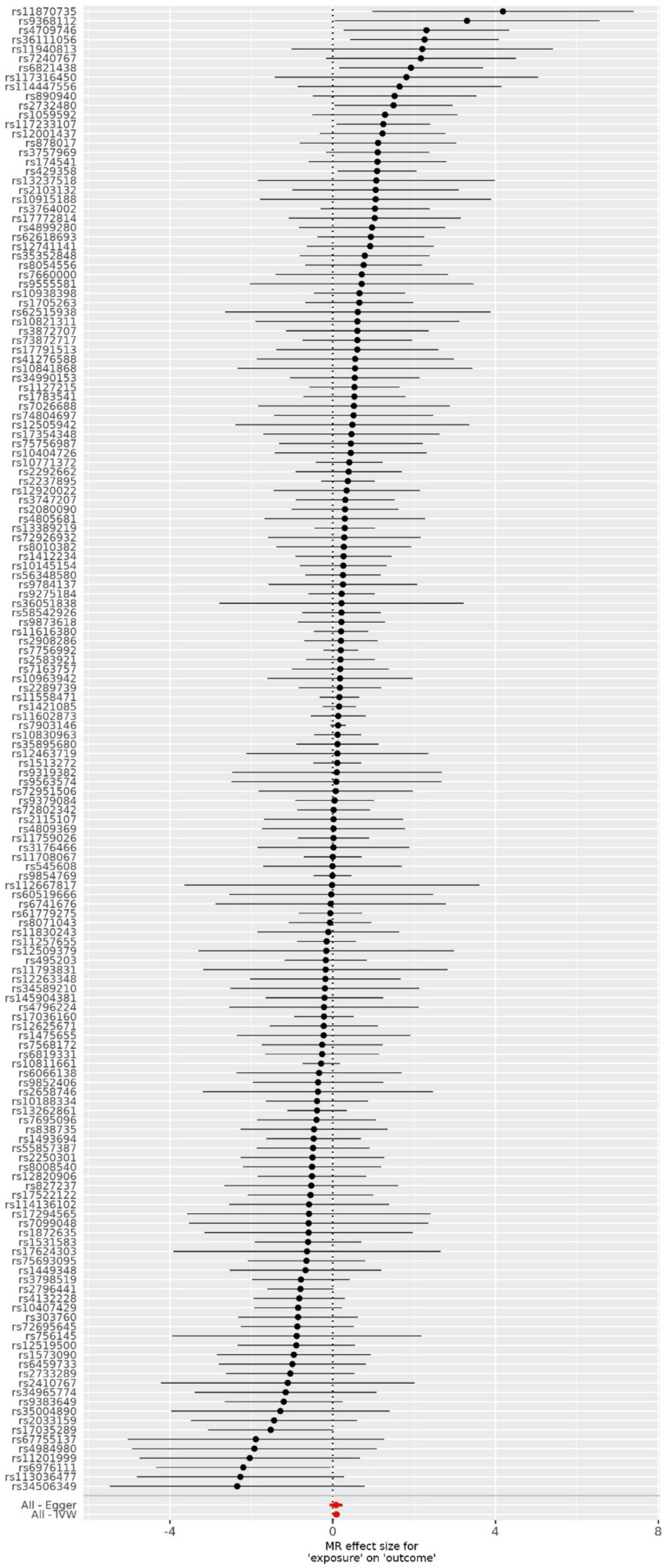

Supplementary Figure 12: Funnel plots showing point estimates as the exposures of interest; Diabetes as exposure. PD risk and progression as outcomes.

PD risk as outcome

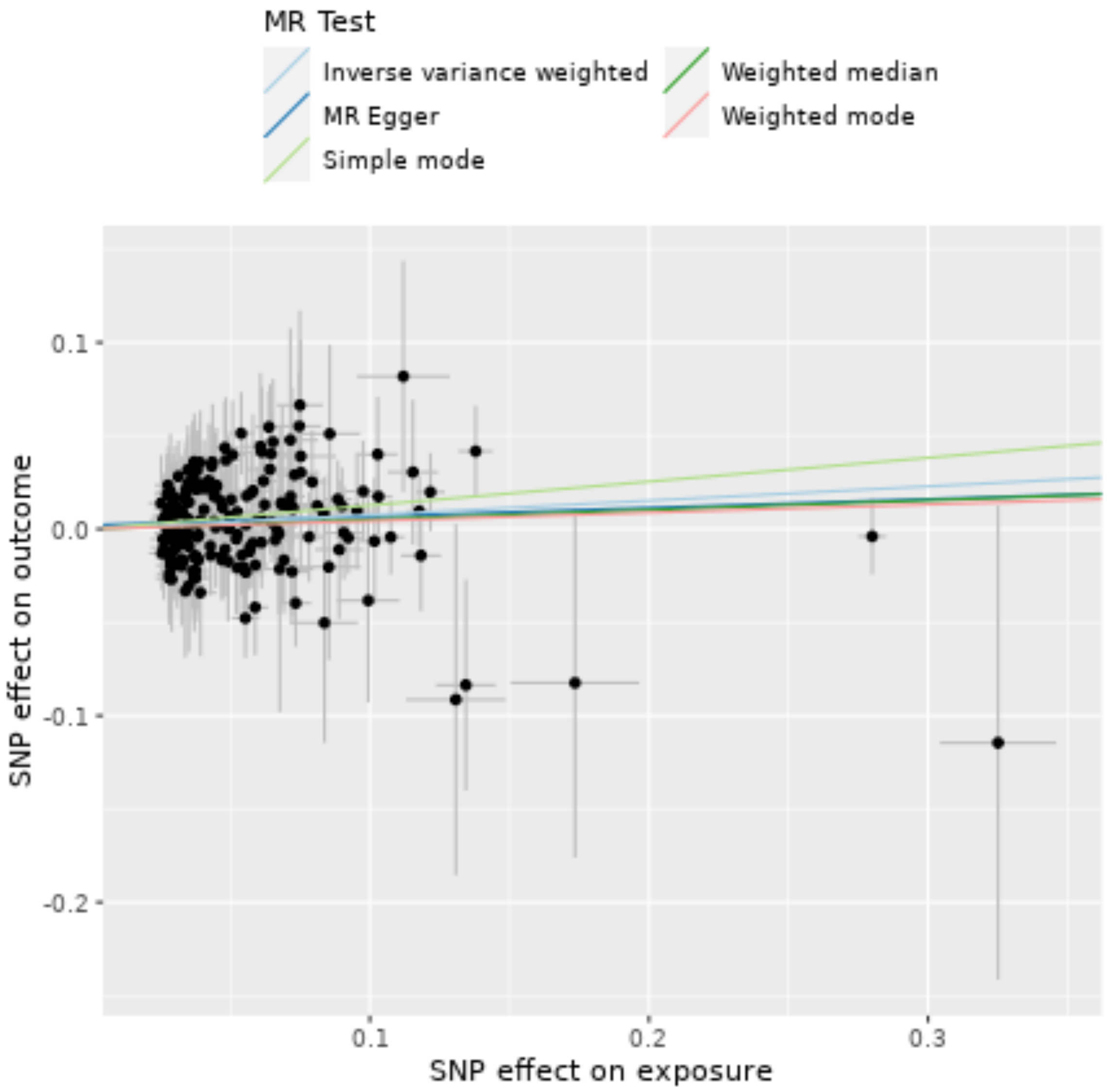

MMSE in PD as outcome

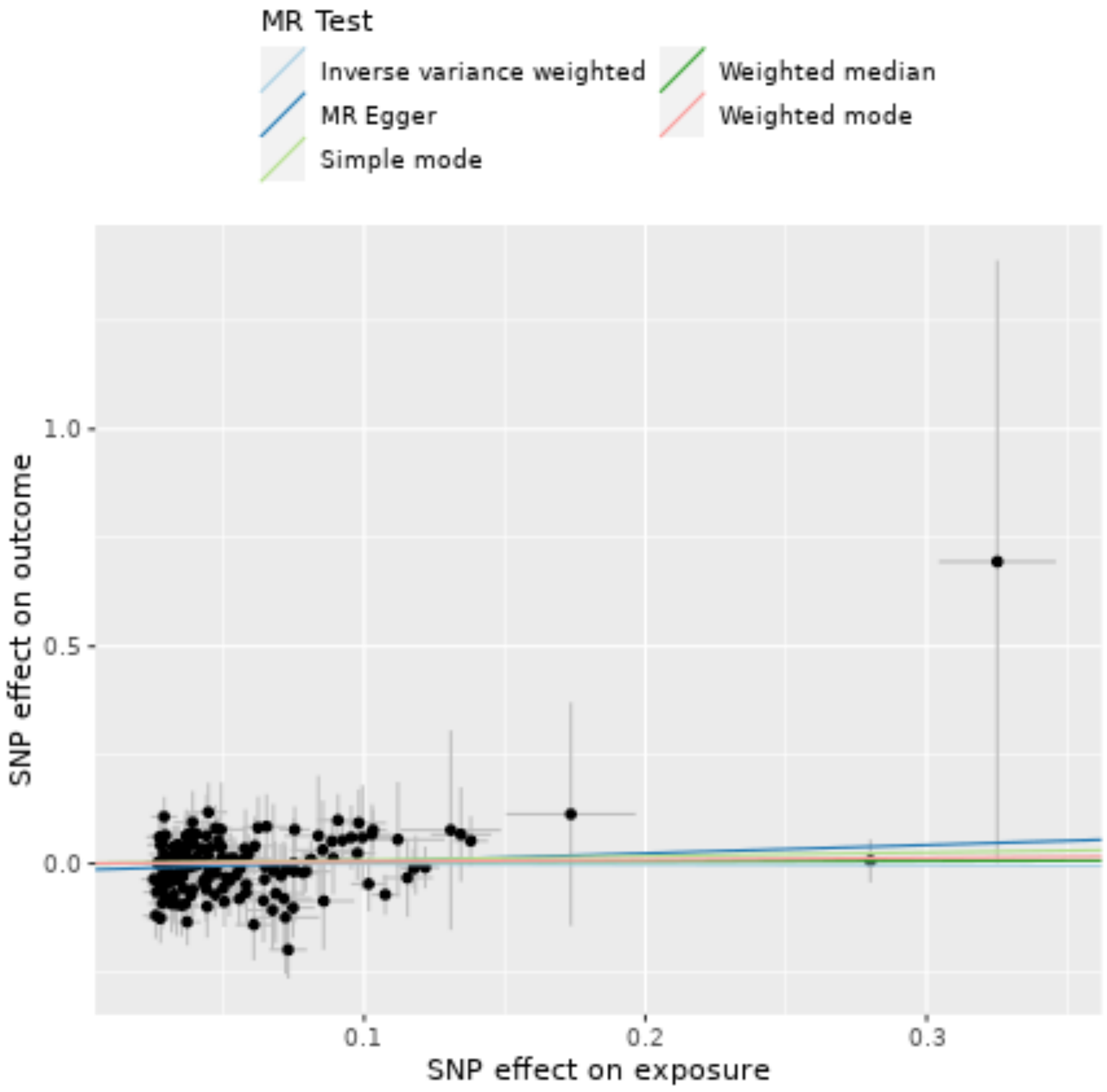

MoCA in PD as outcome

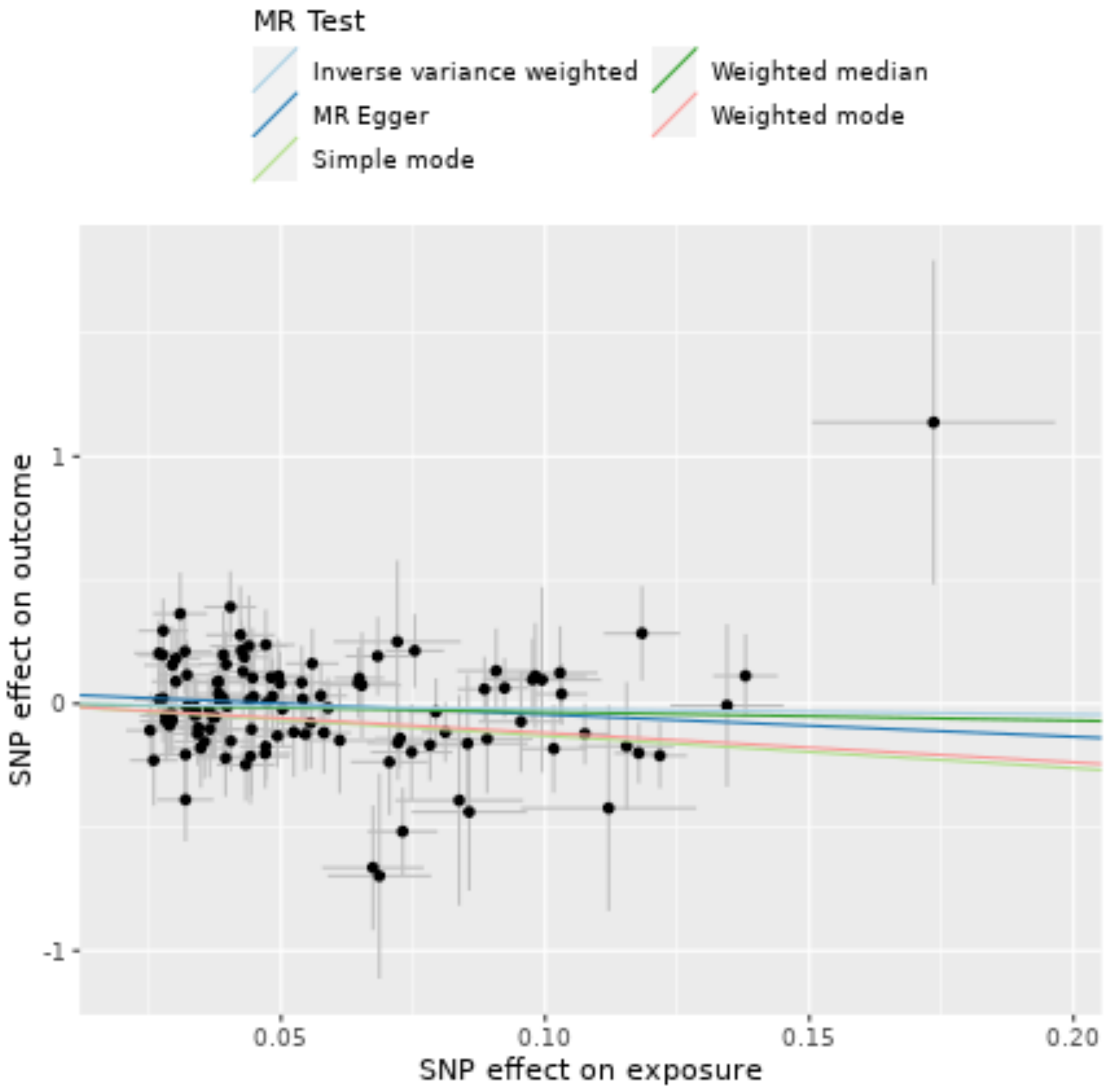

SEADL in PD as outcome

UPDRS3 in PD as outcome

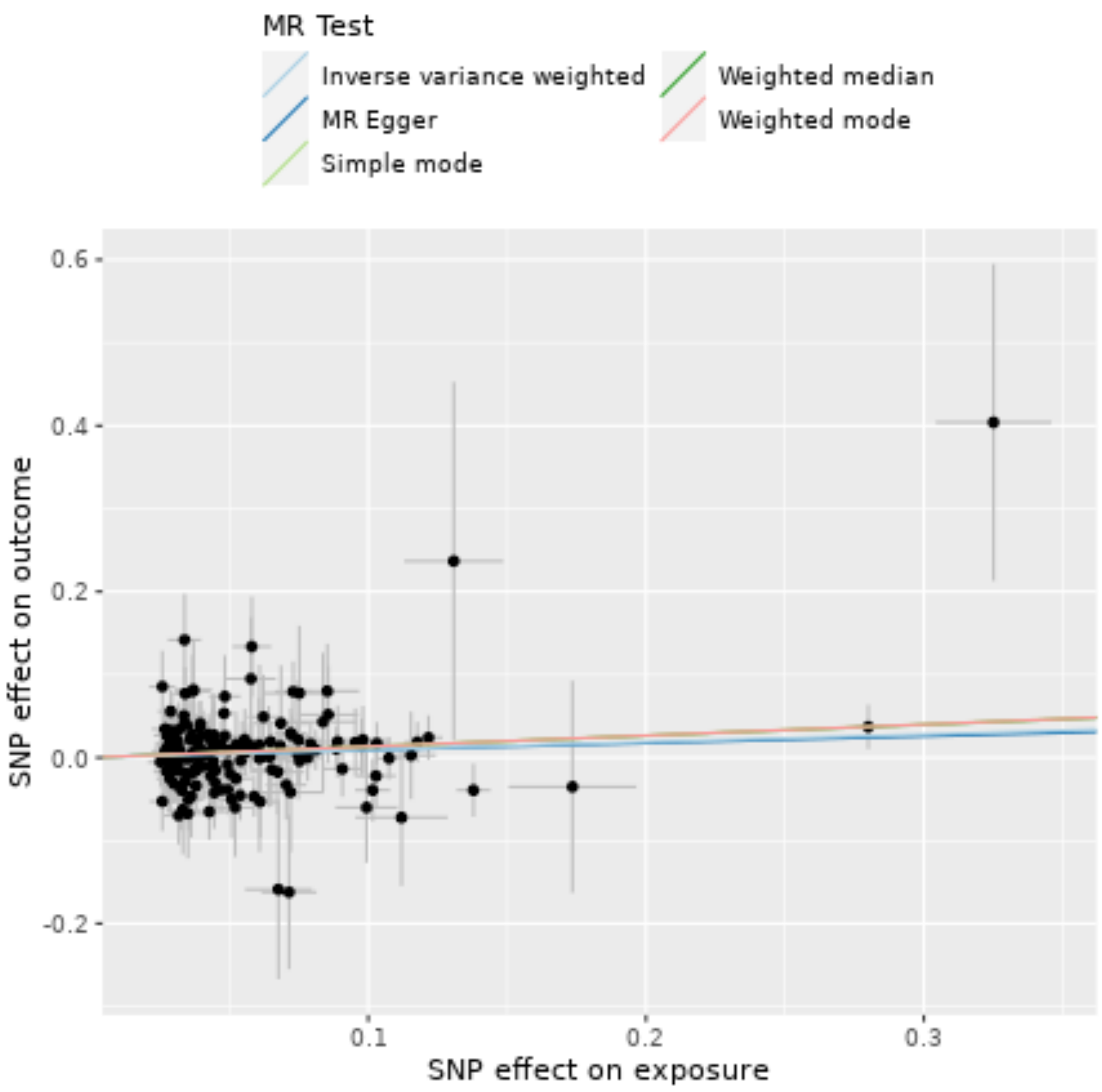

Supplementary Figure 13: Funnel plots evaluated the presence of possible heterogeneity across the estimates. Diabetes as exposure. PD risk and progression as outcomes.

PD risk as outcome

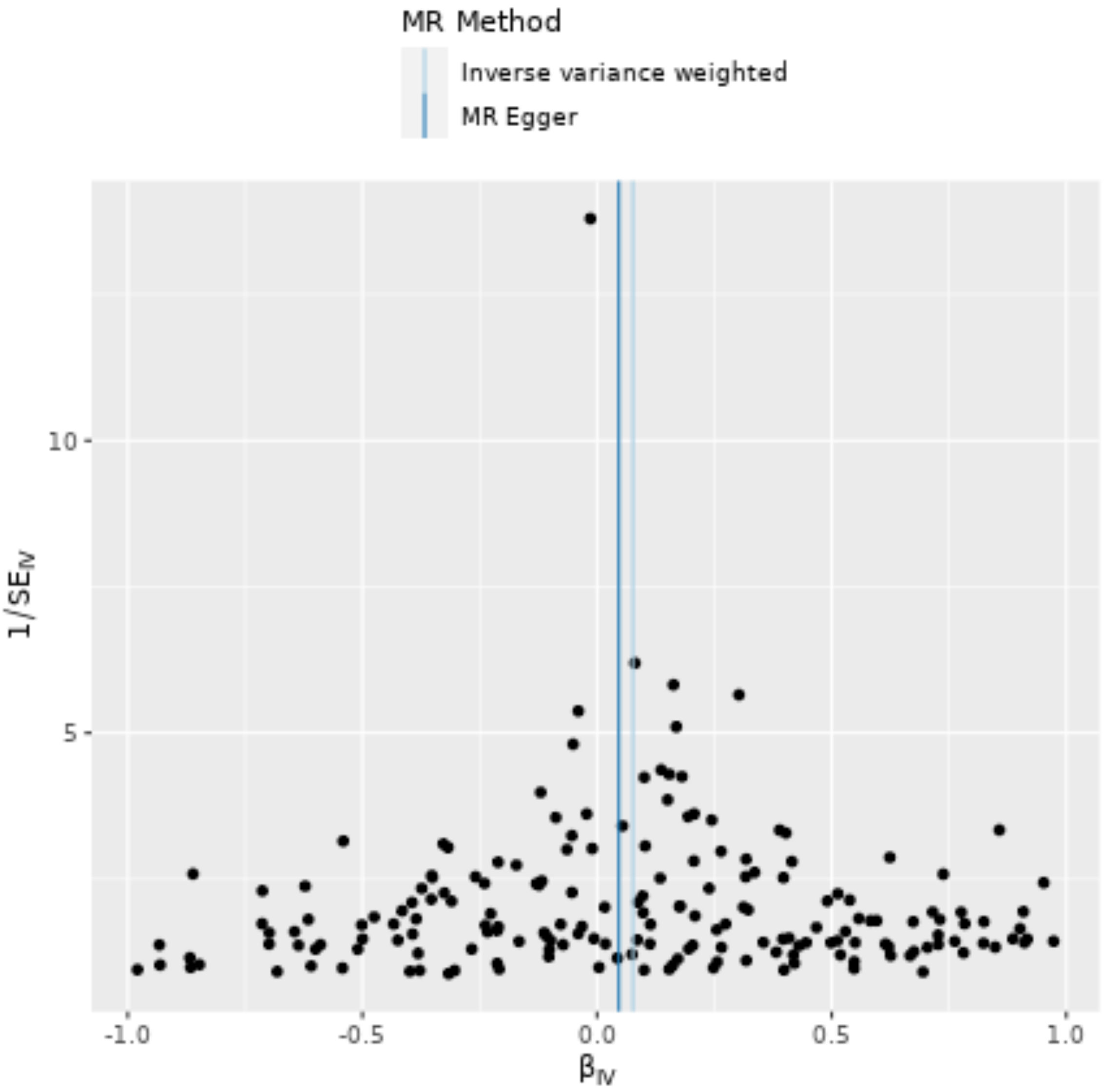

MMSE in PD as outcome

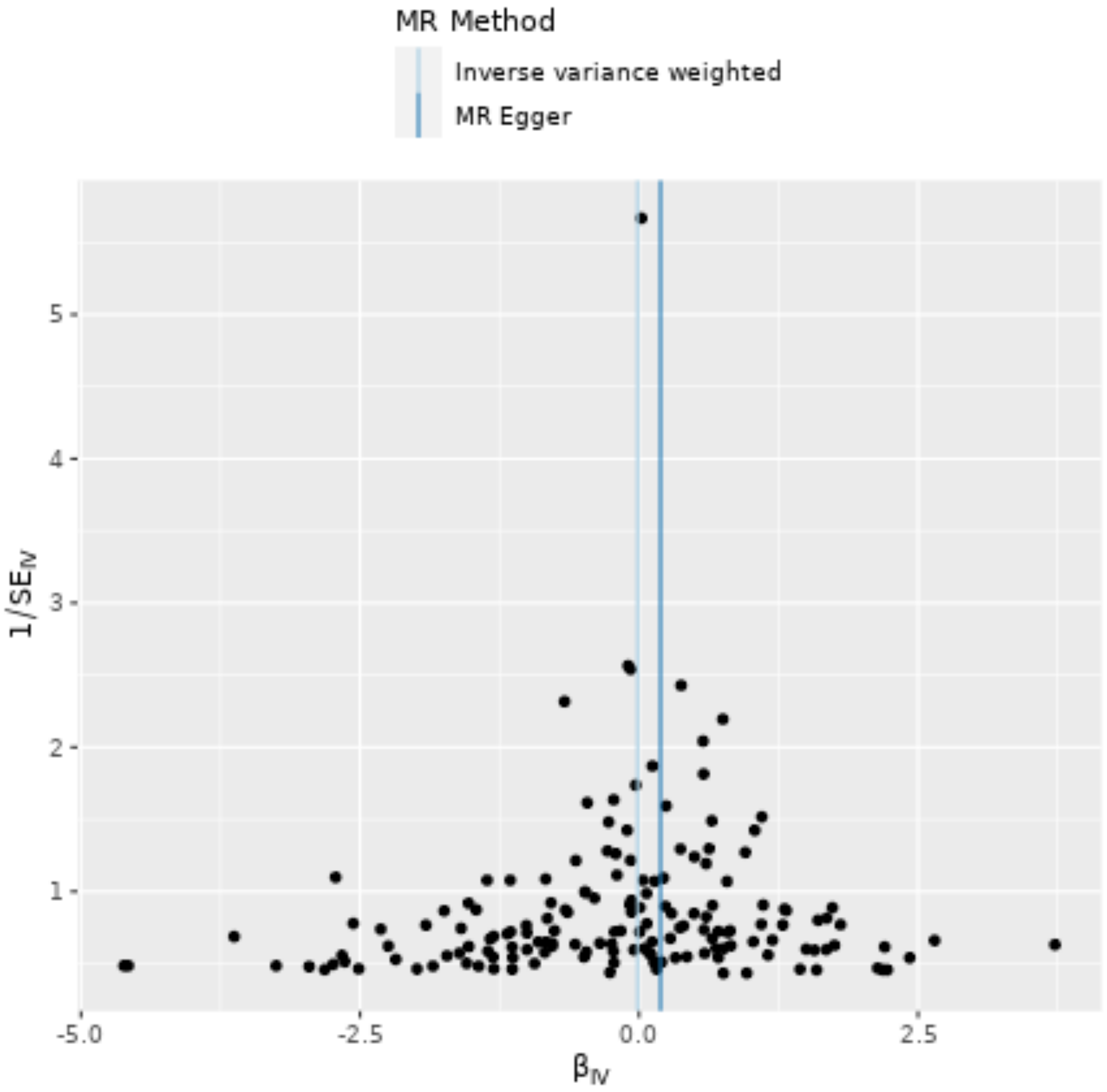

MoCA in PD as outcome

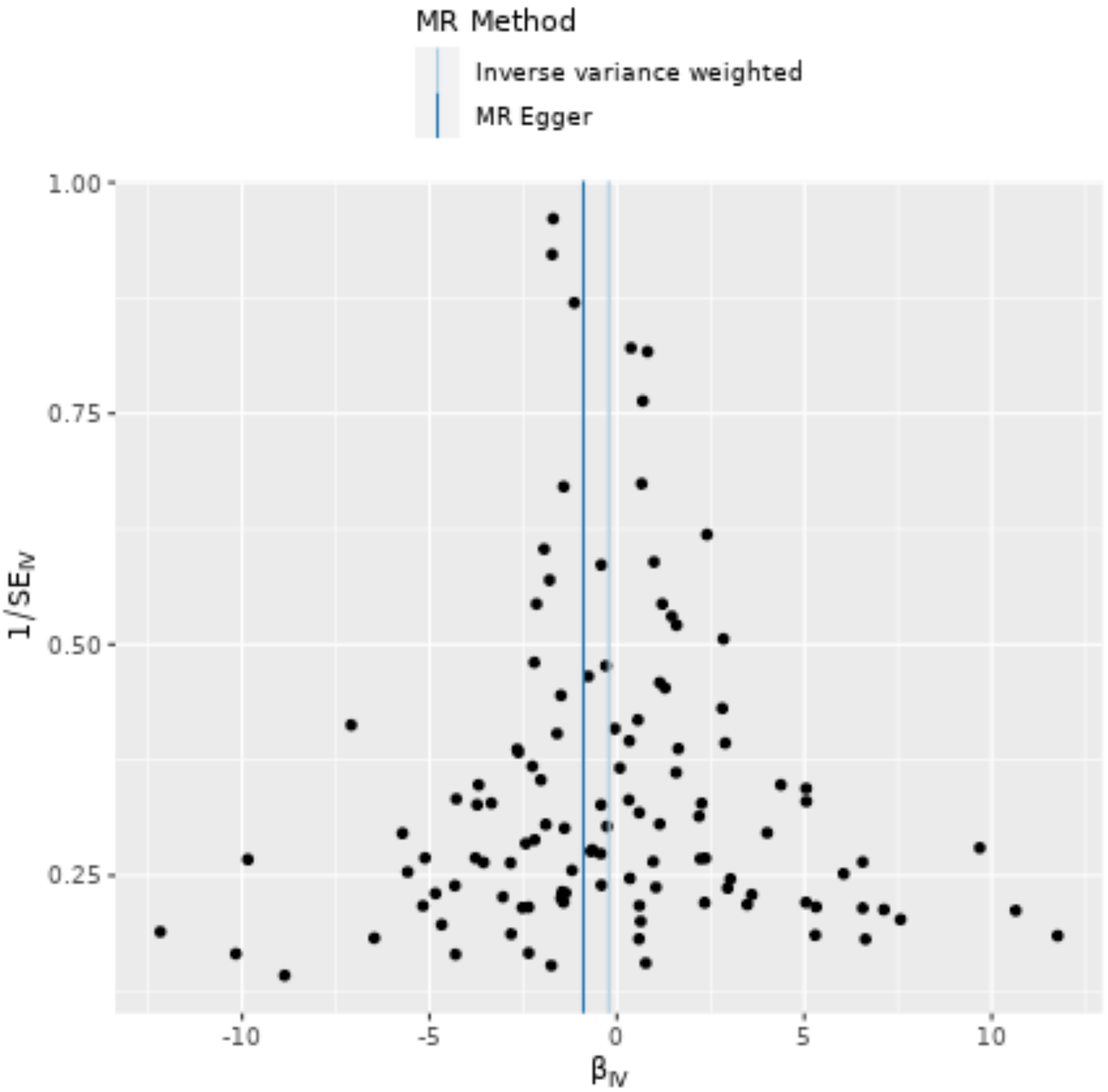

UPDRS3 in PD as outcome

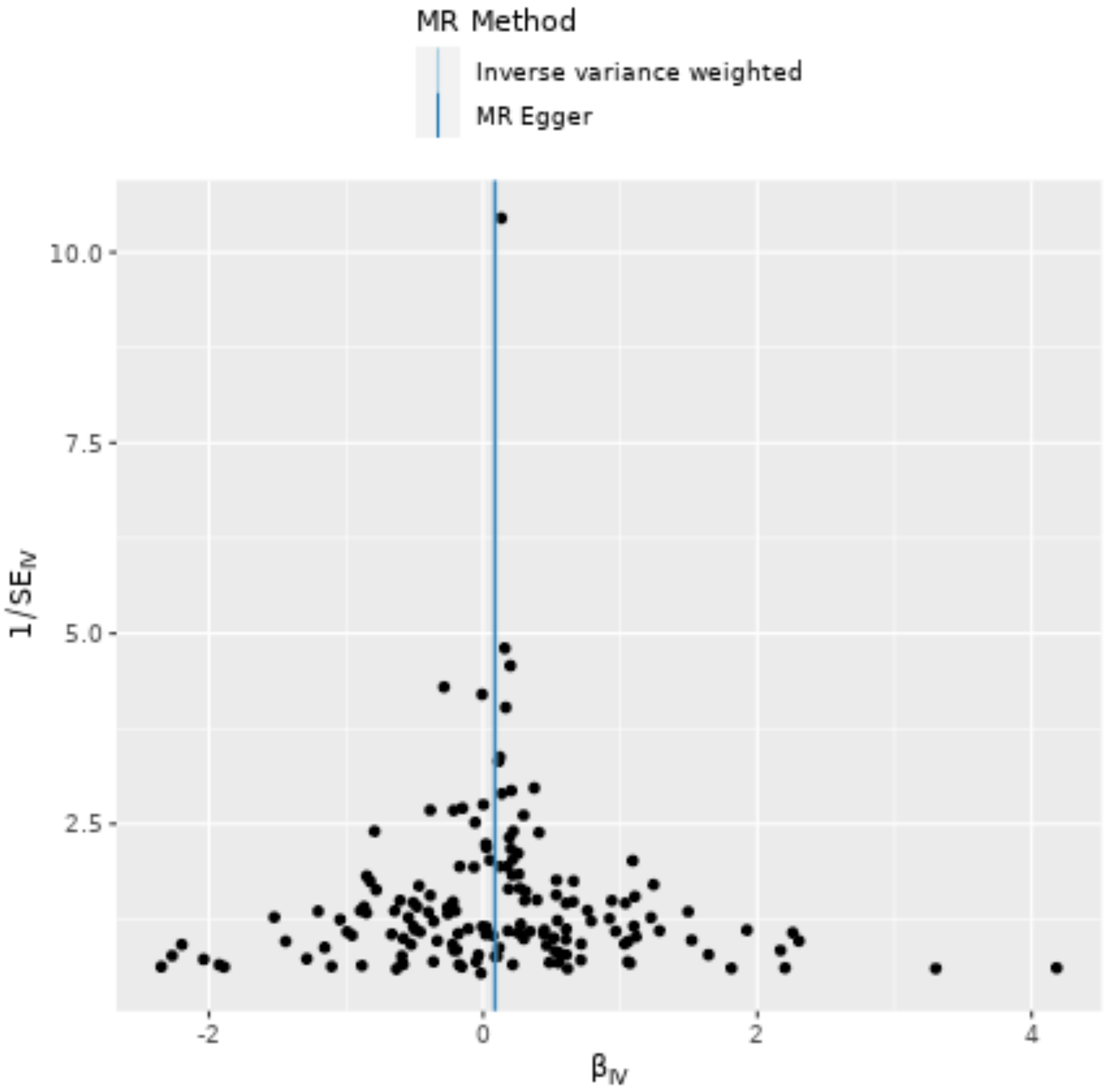

Supplement: Supplementary Information [file NIHMS1794654-supplement-Supplementary_Information.pdf]
